# Handlebody decompositions of three-manifolds and polycontinuous patterns

**DOI:** 10.1098/rspa.2022.0073

**Published:** 2022-04

**Authors:** N. Sakata, R. Mishina, M. Ogawa, K. Ishihara, Y. Koda, M. Ozawa, K. Shimokawa

**Affiliations:** ^1^ Department of Mathematics, Saitama University, Saitama 338-8570, Japan; ^2^ Faculty of Education, Yamaguchi University, Yamaguchi 753-8511, Japan; ^3^ Department of Mathematics, Hiroshima University, Hiroshima 739-8511, Japan; ^4^ Department of Natural Sciences, Faculty of Arts and Sciences, Komazawa University, Tokyo 154-8525, Japan; ^5^ Department of Mathematics, Ochanomizu University, Tokyo 112-8610, Japan

**Keywords:** three-manifold, handlebody decomposition, polycontinuous pattern

## Abstract

We introduce the concept of a handlebody decomposition of a three-manifold, a generalization of a Heegaard splitting, or a trisection. We show that two handlebody decompositions of a closed orientable three-manifold are stably equivalent. As an application to materials science, we consider a mathematical model of polycontinuous patterns and discuss a topological study of microphase separation of a block copolymer melt.

## 1. Introduction

A *Heegaard splitting* is a decomposition of a closed orientable three-manifold into two handlebodies of the same genus. It is well known that every closed orientable three-manifold admits a Heegaard splitting. By the Reidemeister–Singer theorem [1,2], two Heegaard splittings of a given three-manifold are stably equivalent, i.e. isotopic after a finite number of stabilizations.

Many generalizations of Heegaard splittings have been investigated. Gómez-Larrañaga [3] studied orientable three-manifolds decomposed into three solid tori. Coffey and Rubinstein analysed orientable three-manifolds built from three $\pi_{1}$-injective handlebodies [4]. In [5], Koenig considered a *trisection* of a closed orientable three-manifold, which is an embedded branched surface decomposing the manifold into three handlebodies with connected pairwise intersections. Koenig introduced the notion of stabilization for a trisection and showed an analogue of the Reidemeister–Singer theorem for trisections of three-manifolds.

In this paper, we consider a generalization of all of the above. We define a *handlebody decomposition* to be a decomposition of a closed orientable three-manifold into a finite number of handlebodies (see definition 2.1 for the detailed definition). We will also introduce *stabilizations* for handlebody decompositions and show an analogue of the Reidemeister–Singer theorem for handlebody decompositions (see theorem 3.5).

**Figure 1.**
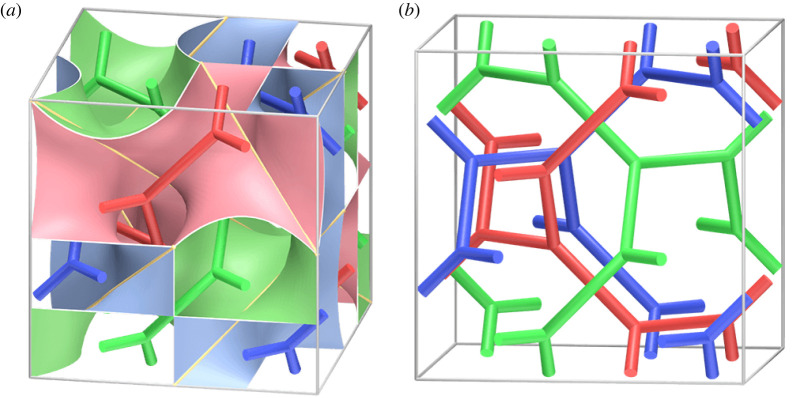
A tricontinuous pattern and an entangled network. (Online version in colour.)

The primary motivation of this study comes from materials science. We are interested in the characterization of *bicontinuous patterns*, *tricontinuous patterns* and *polycontinuous patterns* of microphase separation of a block copolymer melt (see §6b). See [6,7] for related research. A mathematical model of a bicontinuous (resp. tricontinuous or polycontinuous) pattern is a triply periodic non-compact surface (resp. tribranched surface or polyhedron) embedded in $R^{3}$ that divides it into two (resp. three or a finite number of) possibly disconnected submanifolds as shown in figure 1 (see definition 5.8 for more details). We are particularly interested in the case where the submanifolds are the open neighbourhood of networks.

If a bicontinuous pattern is triply periodic, then by considering the quotient of the action, the pattern induces a Heegaard splitting of the three-dimensional torus $T^{3}$ (see remark 7.1). If a polycontinuous pattern is triply periodic and satisfies suitable conditions, then it corresponds to a handlebody decomposition of $T^{3}$ (corollary 5.12). Hence a characterization of handlebody decompositions of $T^{3}$ gives that of triply periodic polycontinuous patterns. The Reidemeister–Singer-type theorem of polycontinuous patterns (corollary 6.3) follows from that of handlebody decompositions of $T^{3}$. This point of view allows us to explain how two polycontinuous patterns are related, which will be discussed in §6b.

This paper is organized as follows. In §2, we define a handlebody decomposition of a three-manifold. In §3, we introduce several types of stabilization operations of handlebody decompositions and prove an analogue of the Reidemeister–Singer theorem for them. In §4, we particularly focus on decompositions of three-manifolds into three handlebodies. In §5, we study a mathematical model of polycontinuous patterns. We define polycontinuous patterns and, more generally, net-like patterns. The correspondence between triply periodic net-like patterns and handlebody decompositions of $T^{3}$ is given. In §6, we discuss stabilizations of net-like patterns. We also present how this research relates to the subject of materials science. In §7, we give characterizations of net-like patterns.

## 2. Handlebody decompositions of three-manifolds

We work in the piecewise linear category throughout this paper.

**Figure 2.**
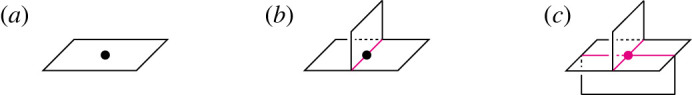
A neighbourhood of each point of a simple polyhedron. (*a*) A non-singular point, (*b*) a triple point and (*c*) a vertex. (Online version in colour.)

By a *two-dimensional polyhedron* P, we mean the underlying space of a non-collapsible locally finite two-dimensional complex such that the link of each vertex contains no isolated vertices. A connected component of the set of points of P having neighbourhoods homeomorphic to discs is called a *sector*. The set of all points not contained in the sectors is called its *singular graph*. A two-dimensional polyhedron P is said to be *simple* if, after giving a structure of a complex in a suitable way, the link of each point in P is homeomorphic to one of the three models shown in figure 2. A point whose link is homeomorphic to the model in figure 2*c* is called a *vertex* of its singular graph. See Matveev [8] for more details.

**Definition 2.1 (Handlebody decomposition).**

Let M be a closed, connected, orientable three-manifold and P a connected compact two-dimensional polyhedron embedded in M. We call $\left(H_{1},H_{2},\ldots ,H_{n};P\right)$ a *type-$\left(g_{1},g_{2},\ldots ,g_{n}\right)$ handlebody decomposition* of M if $M\setminus P=\overset{n}{\underset{i=1}{⨆}}H_{i}$, where $H_{i}$ is the interior of a handlebody of genus $g_{i}$. The polyhedron P is called a *partition* for the decomposition. A handlebody decomposition is said to be *proper* if there is no simple closed curve in M∖B that intersects a sector of P transversely once, where B is the singular graph of P. A handlebody decomposition is said to be *simple* if its partition is a simple polyhedron.

**Remark 2.2.**

(1) Let $\left(H_{1},H_{2},\ldots ,H_{n};P\right)$ be a type-$\left(g_{1},g_{2},\ldots ,g_{n}\right)$ handlebody decomposition of M, and $W_{i}$ a handlebody of genus $g_{i}$ for i=1,2,…,n. Then there exists a continuous map $\iota_{i}:W_{i}\to M$ such that the restriction of $\iota_{i}$ to the interior of $W_{i}$ is a homeomorphism to $H_{i}$. Then we have $\iota_{i}(W_{i})\cap P=\iota_{i}(\partial W_{i})\cap P$. Suppose that the handlebody decomposition is proper. Then for the closure F of each sector, there exists a pair of handlebodies $\left(W_{i},W_{j}\right)$ (i≠j) such that $F\subset \iota_{i}(\partial W_{i})\cap \iota_{j}(\partial W_{j})$. We denote the union of all such surfaces F by $F_{ij}$. (Note that $F_{ij}=F_{ji}$.)

(2) In general, $\iota_{i}$ may not be injective on the singular graph of P. If the decomposition is simple and proper, then $\iota_{i}$ is a homeomorphism.

The notion of handlebody decompositions generalizes both Heegaard splittings [9] and trisections [5] of closed orientable three-manifolds. In fact, a simple proper handlebody decomposition with n=2 is nothing but a Heegaard splitting, while that with n=3, where each $F_{ij}$ is connected, is a trisection. By [10], any closed, connected, three-manifold M admits a simple (non-proper) type-(0) handlebody decomposition. Therefore, it is easily seen that for any sequence $\left(g_{1},\ldots ,g_{n}\right)$ of non-negative integers, there exists a simple (possibly non-proper) type-$\left(g_{1},\ldots ,g_{n}\right)$ handlebody decomposition of M.

## 3. Stable equivalence

This section discusses the stable equivalence of simple proper handlebody decompositions of a three-manifold. We assume that a handlebody decomposition is simple and proper throughout this section. By remark 2.2, for a handlebody decomposition $\left(H_{1},\ldots ,H_{n};P\right)$, there exist handlebodies $W_{1},\ldots ,W_{n}$ and continuous maps $\iota_{1},\ldots ,\iota_{n}$ such that the restriction of each $\iota_{i}$ to the interior of $W_{i}$ is an embedding $\mathrm{int}(W_{i})\to H_{i}$. For simplicity, we regard $H_{i}$ as $\iota_{i}(W_{i})$ and $\partial H_{i}$ as $\iota_{i}(\partial W_{i})$. Then the intersection of $H_{i}$ and $H_{j}$ is a possibly disconnected surface with boundary. We denote it by $F_{ij}$.

### (a) Stabilizations and destabilizations of handlebody decompositions

The following operations for handlebody decompositions are a generalization of the ‘stabilization’ for Heegaard splittings.

**Definition 3.1.**

Let $\left(H_{1},\ldots ,H_{n};P\right)$ be a simple proper type-$\left(g_{1},\ldots ,g_{n}\right)$ handlebody decomposition of a closed, connected, orientable three-manifold M.

**Figure 3.**
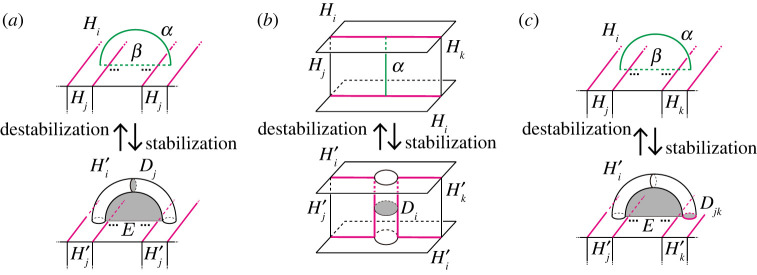
Stabilizations and destabilizations. Red curves represent the singular graphs. Both ends of the arc α of a type-0 stabilization are contained in $F_{ij}$, whereas one end of the arc α of a type-2 stabilization is contained in $F_{ij}$ and the other is in $F_{ik}$ with j≠k. A type-0 stabilization connects two parts of $H_{j}$ by the 1-handle N(α). On the other hand, a new branch locus and a new component $D_{jk}$ of $F_{jk}$ appear after a type-2 stabilization. (*a*) Type-0, (*b*) type-1 and (*c*) type-2. (Online version in colour.)

(0) Take a properly embedded arc α in $H_{i}$, and an arc β in $\partial H_{i}$ such that the endpoints of α lie in the interior of $F_{ij}$, and α is parallel to β in $H_{i}$ relative to the endpoints, i.e. the endpoints of α are equal to that of β, and α∪β bounds a disc in $H_{i}$. Then we get a type-$\left(g_{1}^{\prime },\ldots ,g_{n}^{\prime }\right)$ handlebody decomposition $\left(H_{1}^{\prime },\ldots ,H_{n}^{\prime }\right)$ of M with
  $$g_{l}^{\prime }=\left\{ \begin{array}{ll} g_{i}+1 & \left(l=i\right)\\ g_{j}+1 & \left(l=j\right)\\ g_{l} & \left(l\neq i,j\right) \end{array}\right.\;H_{l}^{\prime }=\left\{ \begin{array}{ll} H_{i}\setminus \mathrm{int}(N(\alpha )) & \left(l=i\right)\\ H_{j}\cup N(\alpha ) & \left(l=j\right)\\ H_{l} & (l\neq i,j), \end{array}\right.$$
  where N(α) and int(N(α)) are a regular neighbourhood of α and its interior in $H_{i}$, respectively. We call this operation a *type-0 stabilization* (along α). Conversely, we assume that there exist properly embedded discs $D_{j}$ of $H_{j}$ and E in $H_{i}$ such that the boundary of $D_{j}$ is in $F_{ij}$, and the boundary of $D_{j}$ intersects that of E transversely exactly one point. Then we can perform the inverse operation of a type-0 stabilization. We call this operation a *type-0 destabilization* (along $D_{j}$). See figure 3*a*.
(1) Take a properly embedded arc α on $F_{jk}$ such that the endpoints of α lie in the boundary of $H_{i}$ for i≠j,k. Then we get a type-$\left(g_{1}^{\prime },\ldots ,g_{n}^{\prime }\right)$ handlebody decomposition $\left(H_{1}^{\prime },\ldots ,H_{n}^{\prime }\right)$ of M with
  $$g_{l}^{\prime }=\left\{ \begin{array}{ll} g_{i}+1 & \left(l=i\right)\\ g_{l} & \left(l\neq i\right) \end{array}\right.\;H_{l}^{\prime }=\left\{ \begin{array}{ll} H_{i}\cup N(\alpha ) & \left(l=i\right)\\ H_{l}\setminus \mathrm{int}(N(\alpha )) & \left(l=j,k\right)\\ H_{l} & (l\neq i,j,k). \end{array}\right.$$
  We call this operation a *type-1 stabilization* (along α). Conversely, if there exists a non-separating disc $D_{i}$ of $H_{i}$ such that the boundary of $D_{i}$ intersects the singular graph of the partition P transversely exactly two points, then we can perform the inverse operation of a type-1 stabilization. We call this operation a *type-1 destabilization* (along $D_{i}$). See figure 3*b*.
(2) Take two points on the interior of $F_{ij}$ and that of $F_{ik}$ for j≠k, and we connect the points by a properly embedded arc α in $H_{i}$. Let β be an arc in $\partial H_{i}$ such that α is parallel to β. Then we get a type-$\left(g_{1}^{\prime },\ldots ,g_{n}^{\prime }\right)$ handlebody decomposition $\left(H_{1}^{\prime },\ldots ,H_{n}^{\prime }\right)$ of M with
  $$g_{l}^{\prime }=\left\{ \begin{array}{ll} g_{i}+1 & \left(l=i\right)\\ g_{l} & \left(l\neq i\right) \end{array}\right.\;H_{l}^{\prime }=\left\{ \begin{array}{ll} H_{i}\setminus \mathrm{int}(N(\alpha )) & \left(l=i\right)\\ H_{j}\cup N(\alpha ) & \left(l=j\right)\\ H_{l} & (l\neq i,j). \end{array}\right.$$
  We call this operation a *type-2 stabilization* (along α). Conversely, if there exists a disc component $D_{jk}$ of $F_{jk}$ whose boundary intersects a properly embedded non-separating disc in $H_{i}$ transversely once, then we can perform the inverse operation of a type-2 stabilization. We call this operation a *type-2 destabilization* (along $D_{jk}$). See figure 3*c*.

**Remark 3.2.**

Consider a type-$\left(g_{1},g_{2},\ldots ,g_{n}\right)$ handlebody decomposition of a closed, connected, orientable three-manifold M with 3≤n. For every $g_{i}\leq g_{i}^{\prime }$, we can obtain a type-$\left(g_{1}^{\prime },g_{2}^{\prime },\ldots ,g_{n}^{\prime }\right)$ handlebody decomposition of M by performing type-1 stabilizations repeatedly in a suitable way.

**Definition 3.3.**

A handlebody decomposition is said to be *stabilized* if it is obtained from another handlebody decomposition by a stabilization.

When n=2, a type-0 stabilization is nothing but a stabilization of Heegaard splittings. In electronic supplementary material, we discuss the independence of these stabilizations.

### (b) Stable equivalence theorem

This subsection will generalize Koenig’s argument [5] on the stable equivalence of decompositions. We first recall the following operations on simple polyhedra embedded in a closed orientable three-manifold introduced by Matveev [8] and Piergallini [11] under our setting.

**Definition 3.4.**

Let P be the partition of a handlebody decomposition of M.

**Figure 4.**
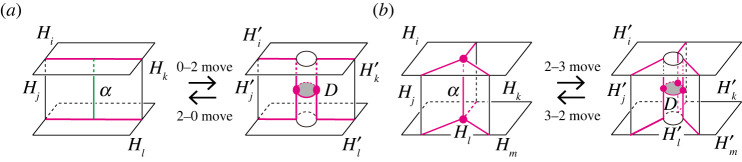
Moves on a handlebody decomposition. As the arc α of a 0-2 (resp. 2-3) move connects $H_{i}$ and $H_{l}$, the 0-2 (resp. 2-3) move produces a new component D of $F_{il}$. The boundary ∂D is contained in the singular graph and has two (resp. three) vertices. A 0-2 move increases the number of vertices by two and a 2-3 move increases by one. (*a*) 0-2 move and 2-0 move and (*b*) 2-3 move and 3-2 move. (Online version in colour.)

(1) Let α be a properly embedded arc in $F_{jk}$. A modification of P in a neighbourhood of α, as in figure 4*a*, is called a *0-2 move along α*. By this operation, the number of vertices of P increases by two, and a new disc component appears in $F_{il}$. Conversely, we can perform the inverse operation of 0-2 move along a disc component, D, of $F_{il}$. We call the operation a *2-0 move along D*. By this operation, the number of vertices of P decreases by two, and the disc component is removed from $F_{il}$.
(2) Let α be an edge of the singular graph of P. A modification of P in a neighbourhood of α, as in figure 4*b*, is called a *2-3 move along α*. By this operation, the number of vertices of P increases by one, and a new disc component appears in $F_{im}$. Conversely, we can perform the inverse operation of 2-3 move along a disc component, D, of $F_{im}$. We call the operation a *3-2 move along D*. By this operation, the number of vertices of P decreases by one, and the disc component is removed from $F_{im}$.

We note that the above moves do not change the topological type of each handlebody of a decomposition.

We say that two handlebody decompositions $\left(H_{1},H_{2},\ldots ,H_{n};P\right)$ and $\left(H_{1}^{\prime },H_{2}^{\prime },\ldots ,H_{n}^{\prime };P^{\prime }\right)$ of a closed orientable three-manifold M are *equivalent* if there exists an ambient isotopy of M that moves P to $P^{\prime }$ and each $H_{i}$ to $H_{i}^{\prime }$ (i=1,…,n) simultaneously.

**Theorem 3.5.**

*Let* $H=(H_{1},H_{2},\ldots ,H_{n};P)$ *and* $H^{\prime }=(H_{1}^{\prime },H_{2}^{\prime },\ldots ,H_{n}^{\prime };P^{\prime })$ *be simple proper handlebody decompositions of a closed orientable three-manifold* M. *Then* H *and* $H^{\prime }$ *are equivalent after applying* 0-2, 2-0, 2-3 *moves and types*-0 *and* -1 *stabilizations finitely many times*.

**Proof.**

Set $F_{ij}=H_{i}\cap H_{j}$ and $F_{ij}^{\prime }=H_{i}^{\prime }\cap H_{j}^{\prime }$ as in remark 2.2. We will prove the theorem in the following steps.

Step 0. In the case of n≥4, we perform 0-2, 2-0, 2-3 moves and type-1 stabilization appropriately until it holds $F_{ij}=\emptyset$ for any 3≤i<j≤n. Then P becomes a simple polyhedron without vertices.
Step 1. For each j∈{3,…,n}, we deform $F_{2j}$ into a disc by type-1 stabilizations. Then, $\left(H_{1},(H_{2}\cup \cdots \cup H_{n})\right)$ is a Heegaard splitting. By applying the same process for $H^{\prime }$, $\left(H_{1}^{\prime },(H_{2}^{\prime }\cup \cdots \cup H_{n}^{\prime })\right)$ becomes a Heegaard splitting, so by the Reidemeister–Singer theorem, we have $H_{1}=H_{1}^{\prime }$ after applying type-0 stabilizations.
Step 2. For each j∈{3,…,n}, we deform $F_{1j}$ into a disc by type-1 stabilizations. We denote by $S_{1}$ the surface $F_{12}$ at this stage, and keep it throughout the steps hereafter.
Step 3. We cover $H_{1}$ along $S_{1}$ with $H_{3}$ by type-1 stabilizations. Then it holds that $H_{3}=H_{3}^{\prime }$ after handle slides.
Step i. (4≤i≤n) We cover $H_{i-1}$ along $S_{1}$ with $H_{i}$ by 0-2, 2-0 moves and type-1 stabilizations. Then it holds that $H_{i}=H_{i}^{\prime }$ after handle slides.

If n=3, after performing the operations described in the first half of Step 0, the decompositions H and $H^{\prime }$ become trisections. Then, they are equivalent by using Koenig’s theorem. Hence, in this proof, we assume that n≥4.

**Step 0.** Put $J=\{(i,j)|3\leq i<j\leq n,\;F_{ij}\neq \emptyset \}$. Let (i,j) be the minimum element of J in the lexicographical order. First, we change $F_{ij}$ to be connected if it is disconnected as follows. Take an arc properly embedded in the closure of $\partial H_{i}\setminus F_{ij}$ that connects different components of $F_{ij}$. If the arc is contained in some $F_{ik}$, a type-1 stabilization along the arc decreases the number of components by one. Otherwise, we can perform a type-1 stabilization after 0-2 moves along the arc to decrease the number of components. Hence, by repeatedly applying this process finitely many times, we may assume that $F_{ij}$ is connected.

Next, take mutually disjoint arcs properly embedded in $F_{ij}$ so that they cut open $F_{ij}$ into a disc. We perform either a type-1 stabilization or a 0-2 move along each of the arcs according to whether both ends of the arc lie in $\partial H_{k}$ for k≠i,j or not. Then $F_{ij}$ becomes a disc. Since P gives the simple proper handlebody decomposition H, the boundary $\partial F_{ij}$ has either at least two vertices or no vertex of P.

**Figure 5.**
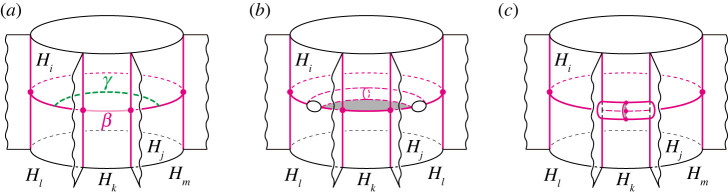
(*a*) The disc sector $F_{ij}$. (*b*) Performing a type-1 stabilization along γ. We can perform a 2-0 move along the greyed region. (*c*) Performing 2-3 move along β. (Online version in colour.)

Suppose $F_{ij}$ is a disc and $\partial F_{ij}$ has more than two vertices. Let β be a sub-arc of $\partial F_{ij}$ cut off by the vertices, γ a properly embedded arc in $F_{ij}$ parallel to β, and $H_{k}$ (k=1,2, or j<k) the handlebody with $\beta \subset \partial H_{k}$ (figure 5*a*). We perform either a 2-0 move along β after a type-1 stabilization along γ or a 2-3 move along β according to whether there exists a different handlebody $H_{l}$ (l=1,2 or j<l) from $H_{k}$ with $\partial \beta \subset H_{k}\cap H_{l}$ or not (figure 5*b*,*c*). Each operation reduces the number of vertices in $\partial F_{ij}$ by two or one. By continuing this process, the number of vertices in $\partial F_{ij}$ can be reduced to two. Then we have $F_{ij}=\emptyset$ after performing a 2-0 move on $F_{ij}$.

Suppose $F_{ij}$ is a disc and $\partial F_{ij}$ has no vertices. There is a handlebody $H_{k}$ (k=1,2, or j<k) with $\partial F_{ij}\subset H_{k}$. In other words, $F_{ik}$ and $F_{jk}$ share their boundary components in $\partial F_{ij}$. Since P is connected, at least one of $F_{ik}$ and $F_{jk}$ has another boundary component. If $F_{ik}$ shares another boundary component with $F_{il}$ (and $F_{kl}$), we take an arc in $F_{ik}$ which connects $\partial F_{ij}$ and $\partial F_{il}$. Then we can remove $F_{ij}$ by a 2-0 move after applying a 0-2 move along the arc. A disc component of $F_{jl}$ arises in this operation. It follows that l>j or l=1,2, as (i,l) is greater than (i,j) in the lexicographical order by the minimality of (i,j). If $F_{jk}$ shares another boundary component with $F_{jl}$ (and $F_{kl}$), similarly, we can remove $F_{ij}$ by a 2-0 move after applying a 0-2 move. In this case, there is a possibility that $F_{il}$ changes to a disc from the empty set by this operation with 3≤l<j. This implies that the minimal element of J varies from (i,j) to (i,l). In such a case, we take an oriented arc in P from a point in $\partial F_{ij}$ to a point in $\partial H_{1}$ or $\partial H_{2}$. Then we can remove $F_{ij}$, and the minimal element of J increases after successively applying 0-2 and 2-0 moves along the arc from the start to the end.

By repeating the same process, we have J=∅. Namely, $F_{ij}=\emptyset$ for 3≤i<j≤n. Since each vertex of P is contained in four different handlebodies, this condition implies that P has no vertex.

**Step 1.** For each j≥3, we will deform $F_{2j}$ into a disc by applying similar operations in Step 0. Since $\partial H_{j}=F_{1j}\cup F_{2j}$, we may assume that $F_{2j}$ is connected, if necessary, by performing type-1 stabilizations along arcs in $F_{1j}$. Take a maximal set of non-separating arcs properly embedded in $F_{2j}$. By performing type-1 stabilizations along the arcs, $F_{2j}$ becomes a disc. Then $H_{2}\cup \cdots \cup H_{n}$ is a handlebody. By applying the same process for $H^{\prime }$, $H_{2}^{\prime }\cup \cdots \cup H_{n}^{\prime }$ becomes a handlebody. Hence $\left(H_{1},(H_{2}\cup \cdots \cup H_{n})\right)$ and $\left(H_{1}^{\prime },(H_{2}^{\prime }\cup \cdots \cup H_{n}^{\prime })\right)$ are Heegaard splittings of M. By the Reidemeister–Singer theorem, these two Heegaard splittings become equivalent after performing a finite sequence of type-0 stabilizations. In particular, we can assume $H_{1}=H_{1}^{\prime }$.

**Step 2.** Similarly to Step 1, for each j≥3, we can deform $F_{1j}$ into a disc by performing type-1 stabilizations along suitable arcs properly embedded in $F_{1j}$.

**Claim 3.6.**

For i∈{3,…,n}, let $D_{i1},\ldots ,D_{ig_{i}}$ be a complete meridian disc system of $H_{i}$ such that $\partial D_{ij}\subset F_{2i}$ for $j\in \{1,\ldots ,g_{i}\}$. Then there exist disjoint meridian discs $E_{ij}$ (i∈{3,…,n}, $j\in \{1,\ldots ,g_{i}\}$) of $H_{2}$ such that $\partial E_{ij}\subset F_{12}\cup F_{2i}$, $E_{ij}\cap D=E_{ij}\cap D_{ij}$, and $\partial E_{ij}$ intersects $\partial D_{ij}$ transversely in a single point, where D denotes the union $\cup_{i,j}D_{ij}$ of all $D_{ij}$.

**Proof of claim 3.6.**

**Figure 6.**
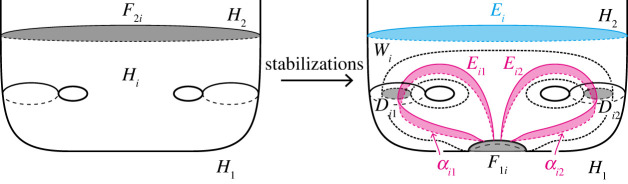
A deformation at Step 2. (Online version in colour.)

According to the deformation of $H_{2}$ at this step, there exist mutually disjoint separating discs $E_{3},\ldots ,E_{n}$ in $H_{2}$ such that each $E_{i}$ cuts off a handlebody $W_{i}$ from $H_{2}$ so that $\left(W_{i},F_{2i}\right)$ is homeomorphic to $\left(F_{2i}\times [0,1],F_{2i}\times \{0\}\right)$. (The union $H_{i}\cup W_{i}$ can be regarded as the handlebody $H_{i}$ at the end of Step 1.) We can take mutually disjoint arcs $\alpha_{i1},\ldots ,\alpha_{ig_{i}}$ properly embedded in $F_{2i}$ so that $\alpha_{ij}\cap D=\alpha_{ij}\cap D_{ij}$, and $\alpha_{ij}$ intersects $D_{ij}$ transversely in a single point. See figure 6. Let $E_{ij}$ be a disc corresponding to $\alpha_{ij}\times [0,1]$ such that $E_{ij}\cap E_{i}=\emptyset$ for each $j\in \{1,\ldots ,g_{i}\}$. Then the assertion holds since $(\partial W_{i}\setminus E_{i})\subset F_{12}\cup F_{2i}$. ▪

Let $S_{1}$ denote the surfaces $F_{12}$ at this stage. Claim 3.6 implies that any one-handle of each handlebody $H_{i}$ (i≥3) can be a local one-handle after a handle slide on $S_{1}$.

**Figure 7.**
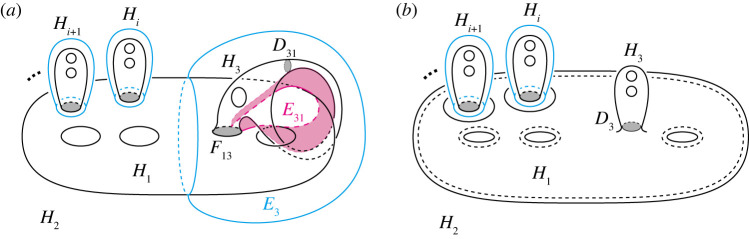
(*a*) Before performing the operation in Step 3. (*b*) After performing the operation. The handlebody $H_{3}$ is a local unknotted handlebody. (Online version in colour.)

**Step 3.** For handle slide of $H_{3}$, we will cover $H_{1}$ along $S_{1}$ with $H_{3}$ by type-1 stabilizations. Take a maximal set of mutually non-parallel, non-boundary parallel arcs properly embedded in $S_{1}$ whose endpoints lie in $\partial H_{3}$. We perform type-1 stabilizations along those arcs. The surface $F_{12}=S_{1}\setminus F_{13}$ becomes the union of n−3 annuli $A_{14},\ldots ,A_{1n}$ such that $A_{1j}\cap H_{j}=\partial F_{1j}=\partial F_{2j}$ for each j∈{4,…,n}. Since all spines of $S_{1}$ are covered by $H_{3}$, $H_{3}$ becomes a local unknotted handlebody after performing handle slides by claim 3.6 (figure 7). Applying the same process for $H^{\prime }$ and arranging genera of $H_{3}$ and $H_{3}^{\prime }$ by performing type-0 stabilizations if necessary, we can assume that $H_{3}=H_{3}^{\prime }$.

According to the deformation of $H_{3}$ at this step, there exists a separating disc $D_{3}$ in $H_{3}$ that cuts off a handlebody $V_{3}$ from $H_{3}$ so that $\left(V_{3},F_{13}\right)$ is homeomorphic to $\left(F_{13}\times [0,1],F_{13}\times \{0\}\right)$. ($H_{3}\setminus V_{3}$ can be regarded as the previous $H_{3}$ at the end of Step 2.) Let $S_{3}$ be the surface $\partial V_{3}\setminus (D_{3}\cup F_{13})$, which is a subsurface of $F_{23}$ and homeomorphic to $S_{1}$.

**Step** *i* **(**4≤i≤n**).** At the beginning of Step i, we may have $S_{3},\ldots ,S_{i-1}$ as subsurfaces of $\partial H_{3},\ldots ,\partial H_{n-1}$, respectively, that are homeomorphic to $S_{1}\subset \partial H_{1}$, and (i−3) annuli $A_{1i},A_{3i},\ldots ,A_{(i-2)i}\subset \partial H_{2}$ between $\partial F_{2i}$ and a component of $\partial S_{i-1}$, where $A_{ji}\subset S_{j}\setminus F_{j(j+1)}$ for each j∈{3,…,i−2}, $A_{3i}\cap S_{1}=A_{3i}\cap A_{1i}=\partial A_{3i}\cap \partial A_{1i}$, and $A_{ji}\cap S_{j-1}=A_{ji}\cap A_{(j-1)i}=\partial A_{ji}\cap \partial A_{(j-1)i}$ for each j∈{4,…,i−2}.

Similar to Step 3, we will cover $H_{i-1}$ along $S_{1}$ with $H_{i}$ by performing handle slides of $H_{i}$. By a 0-2 move and a 2-0 move on $A_{1i}$, $F_{1i}$ extends to $A_{1i}$, and an annulus $F_{3i}$ arises. Continuing the same operation on $A_{3i},\ldots ,A_{(i-2)i}$, $F_{ji}$ (j∈{1,3,…i−2}) becomes the annulus $A_{ji}$. By the same operation as Step 3 on $S_{i-1}$, $S_{i-1}\setminus F_{(i-1)i}$ becomes the union of n−4 annuli including $A_{\left(i-1)(i+1\right)},\ldots ,A_{(i-1)n}$. (In the case of n=4, $F_{34}$ includes $S_{3}$ at Step 4.) By the same argument at Step 3, then, $H_{i}$ can be a local unknotted handlebody after handle slide, and we can assume that $H_{i}=H_{i}^{\prime }$.

When we finish Step n, we have $H_{1}=H_{1}^{\prime },\;H_{3}=H_{3}^{\prime },\ldots ,H_{n}=H_{n}^{\prime }$. Since this automatically implies that $H_{2}=H_{2}^{\prime }$, the proof is completed. ▪

## 4. Handlebody decomposition consisting of three handlebodies

In this section, we provide several results of handlebody decompositions consisting of three handlebodies. We keep assuming that all handlebody decompositions are simple and proper unless otherwise specified.

Section 4a will consider stabilizability on handlebody decompositions containing a three-ball. In [12], Waldhausen showed that any genus-g Heegaard splitting of $S^{3}$ is stabilized for g≥1. On the other hand, Koenig found an infinite family of unstabilized type-(1,2,2) handlebody decompositions of $S^{3}$ (see §6 in [5]). We will show that a closed connected orientable three-manifold not containing a non-separating sphere admits an unstabilized type-(0,0,g) handlebody decomposition, where g is the Heegaard genus of the manifold (proposition 4.2). Furthermore, we will see that almost all lens spaces admit a type-(0,1,2) handlebody decomposition (proposition 4.9). In §4b, we will study handlebody decompositions of the three-dimensional torus $T^{3}$. These decompositions play an important role in *polycontinuous patterns* (§5).

### (a) Handlebody decompositions containing a three-ball

We first introduce the result of Gómez-Larrañaga [3]. That result gave a complete classification of all closed connected three-manifolds that admit handlebody decompositions with small genera.

**Theorem 4.1 ([3, propositions 1–3, theorem 1]).**

*Let* $\left(H_{1},H_{2},H_{3}\right)$ *be a type*-$\left(g_{1},g_{2},g_{3}\right)$ *handlebody decomposition of a closed connected orientable three-manifold* M *with* $g_{1}\leq g_{2}\leq g_{3}$. *We denote by* B *the connected sum of some copies of* $S^{2}\times S^{1}$, *and denote by* L *or* $L_{i}$ *a lens space with non-trivial finite fundamental group. Then the following hold*:

(1) *If all* $g_{i}$ *are equal to* 0, *then* M *is homeomorphic to* $S^{3}$ *or* B. *Conversely*, $S^{3}$ *and* B *admit such a handlebody decomposition*.
(2) *If* $g_{1}=g_{2}=0$ *and* $g_{3}=1$, *then* M *is homeomorphic to* $S^{3}$, B, L *or* B#L. *Conversely, these manifolds admit such a handlebody decomposition*.
(3) *If* $g_{1}=0$ *and* $g_{2}=g_{3}=1$, *then* M *is homeomorphic to* $S^{3}$, B, L, B#L, $L_{1}\#L_{2}$ *or* $L_{1}\#L_{2}\#B$. *Conversely, these manifolds admit such a handlebody decomposition*.
(4) *If all* $g_{i}$ *are equal to* 1, *then* M *is homeomorphic to* $S^{3}$, B, L, B#L, $L_{1}\#L_{2}$, $L_{1}\#L_{2}\#B$, $L_{1}\#L_{2}\#L_{3}$, $L_{1}\#L_{2}\#L_{3}\#B$, S(3) *or* S(3)#B, *where* S(3) *denotes a Seifert fibre space with at most three exceptional fibres. Conversely, these manifolds admit such a handlebody decomposition*.

Let M be a closed orientable three-manifold with a Heegaard splitting $\left(W_{1},W_{2}\right)$ of genus l. Then, we can take l+1 non-separating discs in $W_{1}$ so that they separate $W_{1}$ into two three-balls. Hence, M admits a type-(0,0,l) handlebody decomposition (see [13, example 1.2]). The following proposition classifies such a decomposition.

**Proposition 4.2.**

*Let* M *be a closed, connected, orientable three-manifold of Heegaard genus* g. *Suppose that* M *does not contain a non-separating sphere. Then* M *admits a type*-(0,0,l) *handlebody decomposition if and only if we have* g≤l. *In particular, a type*-(0,0,g) *handlebody decomposition of* M *is unstabilized.*

To prove the above proposition, we first show the following lemma.

**Lemma 4.3.**

*Let* $\left(H_{1},H_{2},H_{3};P\right)$ *be a type*-(0,0,l) *handlebody decomposition of a closed, connected, orientable three-manifold* M. *Suppose that* M *does not contain a non-separating sphere. Then* $\left(H_{1}\cup H_{2},H_{3}\right)$ *is a genus*-l *Heegaard splitting of* M.

**Proof.**

Let $F_{ij}$ denote a surface as in remark 2.2. We show that the surface $F_{12}$ consists of discs. Assume that $F_{12}$ contains a non-disc component S. Then there exists an essential simple loop C in S such that each complementary region of C in $\partial H_{1}≅S^{2}$ contains a connected component of $F_{13}$. Since $H_{1}$ and $H_{2}$ are three-balls, the simple loop C bounds a disc in each of $H_{1}$ and $H_{2}$. Then the union of the two discs is a non-separating disc, which is a contradiction.

Thus, $F_{12}$ consists of only discs. It follows that the union of $H_{1}$ and $H_{2}$ is a handlebody, which implies the assertion. ▪

**Proof of proposition 4.2.**

Let g be the Heegaard genus of a closed, connected, orientable three-manifold M. Then, as explained above, M admits a type-(0,0,g) handlebody decomposition. Then, by remark 3.2, we can obtain a type-(0,0,l) handlebody decomposition of M for each g≤l. Conversely, assume that M admits a type-(0,0,l) handlebody decomposition. By lemma 4.3, this handlebody decomposition induces a Heegaard splitting of genus l. Thus, we have g≤l. This particularly implies that a type-(0,0,g) handlebody decomposition of M is unstabilized. ▪

**Example 4.4.**

An unstabilized type-(0,0,2) handlebody decomposition of $S^{3}$ is constructed as follows. Let $\left(W_{1},W_{2}\right)$ be a genus-2 Heegaard splitting of $S^{3}$. By using [14, §5 and fig. 4], we can take a non-primitive disc triple of $W_{1}$, which separates $W_{1}$ into two three-balls. We denote the three-balls by $H_{1}$ and $H_{2}$, and put $H_{3}=W_{2}$. Then $\left(H_{1},H_{2},H_{3}\right)$ forms a type-(0,0,2) handlebody decomposition of $S^{3}$. Because each component of $F_{12}$ is a non-primitive disc in $W_{1}$, we can see that the boundary of any properly embedded disc in $H_{3}$ transversely intersects the singular graph of the partition in at least six points. Hence we cannot perform a destabilization along any properly embedded discs in $H_{3}$. Therefore, the decomposition is unstabilized.

Next, we will consider the stabilizability of type-(0,1,l) handlebody decompositions.

**Proposition 4.5.**

*Let* M *be a closed, connected, orientable, irreducible three-manifold. Suppose that* M *is not a lens space with non-trivial finite fundamental group. Then, for each* 1≤l, *any type*-(0,1,l) *handlebody decomposition of* M *is stabilized. In fact, such a decomposition is obtained from a type*-(0,0,l) *handlebody decomposition by performing a type*-1 *stabilization*.

**Proof.**

We first assume that $F_{12}$ consists of discs. Then there exists a meridian disc of $H_{2}$ whose boundary intersects $\partial F_{12}$ transversely exactly twice. Hence we can perform a type-1 destabilization along the meridian disc.

In the remainder, we assume that $F_{12}$ has a non-disc component.

**Claim 4.6.**

We have χ(S)≥0 for each component S of $F_{12}$.

**Proof of claim 4.6.**

Suppose that χ(S)<0. Since $S\subset \partial H_{1}≅S^{2}$, the boundary ∂S has at least three components. Then there exists a component c of ∂S such that it is an inessential loop in $\partial H_{2}≅T^{2}$. Hence c bounds a properly embedded disc in $H_{2}$. The closed curve c also bounds a properly embedded disc in $H_{1}$ since $H_{1}$ is a three-ball. Because each complementary region of c in $\partial H_{1}≅S^{2}$ contains a component of $F_{13}$, the two properly embedded discs in $H_{1}$ and $H_{2}$ form a non-separating sphere. This contradicts the irreducibility of M. ▪

**Claim 4.7.**

A core curve of each annulus component of $F_{12}$ is essential in $\partial H_{2}≅T^{2}$.

**Proof of claim 4.7.**

Assume that a core curve C of an annulus component of $F_{12}$ is inessential in $\partial H_{2}$. Then C bounds a properly embedded disc in $H_{2}$, and each complementary region of C intersects $F_{23}$. Since $H_{1}$ is a three-ball, C also bounds a properly embedded disc in $H_{1}$. Hence the two discs form a non-separating sphere. This is a contradiction. ▪

**Claim 4.8.**

The surface $F_{12}$ contains precisely one annulus component.

**Proof of claim 4.8.**

We assume that $F_{12}$ contains two annulus components. Then, by claim 4.7, their core curves, $C_{1}$ and $C_{2}$, are parallel essential loops in $\partial H_{2}≅T^{2}$. Thus, $C_{1}\cup C_{2}$ cobounds a properly embedded annulus in $H_{2}$. Since $H_{1}$ is a three-ball, each of $C_{1}$ and $C_{2}$ bounds a disc in $H_{1}$. Because each complementary region of $C_{1}\cup C_{2}$ in $\partial H_{2}$ intersects $F_{23}$, the union of the annulus and the discs is a non-separating sphere. This is a contradiction.

Let C be a core curve of the annulus component A of $F_{12}$. Then we have $\left[C]=a\mu +b\lambda \in H_{1}(\partial H_{2}\right)$, where a,b∈Z, and μ and λ denote homology classes of a meridian loop and a longitude loop, respectively. Since $H_{1}$ is a three-ball, each component of ∂A bounds a properly embedded disc in $H_{1}$. Then the union S of $\partial H_{2}\setminus \mathrm{int}(A)$ and the two discs is a separating sphere in M. Thus, if b≠0 and ±1, then M has a lens space as a connected summand. However, M is irreducible and not a lens space. Hence we have b=0 or ±1. If b=0, then C bounds a properly embedded disc in $H_{2}$. The curve C also bounds a properly embedded disc in $H_{1}$ since $H_{1}$ is a three-ball. So, the discs form a non-separating sphere, which is a contradiction. Hence we have b=±1. Thus, we can take a meridian disc of $H_{2}$ that intersects the boundary of $F_{12}$ transversely exactly two points. Therefore, we can perform a type-1 destabilization along the meridian disc. ▪

The following proposition implies that the assumption that M is not a lens space in proposition 4.5 is essential.

**Proposition 4.9.**

*Any lens space with non-trivial finite fundamental group admits an unstabilized type*-(0,1,2) *handlebody decomposition*.

**Proof.**

**Figure 8.**
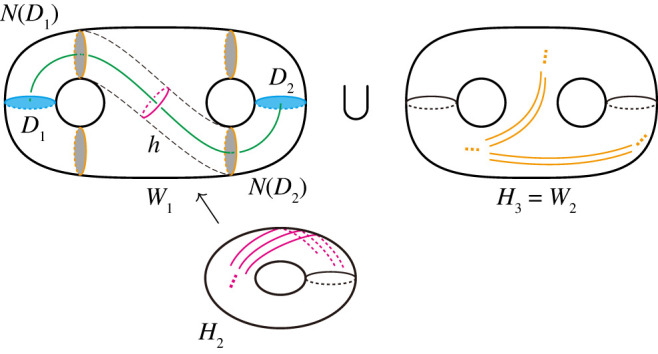
An unstabilized type-(0,1,2) handlebody decomposition of a lens space with non-trivial finite fundamental group. The decomposition consists of three handlebodies $H_{1}=h\cup N(D_{1})\cup N(D_{2})$, $H_{2}$ and $H_{3}=W_{2}$.

Let $\left(W_{1},W_{2}\right)$ be a genus-2 Heegaard splitting of $S^{3}$. Then there exists a pair of non-primitive discs $D_{1}$ and $D_{2}$ in $W_{1}$ as in example 4.4. Note that $B:=N(D_{1};W_{1})\cup N(D_{2};W_{1})\cup W_{2}$ is a three-ball, where $N(D_{1};W_{1})$ and $N(D_{2};W_{1})$ are regular neighbourhoods of $D_{1}$ and $D_{2}$, respectively. We take an unknotted arc δ in the interior of $W_{1}$ that joins $D_{1}$ and $D_{2}$ and intersects them at only its endpoints. Let h be a one-handle attached to sides of each $N(D_{1};W_{1})$ and $N(D_{2};W_{1})$ along δ. Then, h∪B is a solid torus (figure 8). Hence, for any lens space M, there exists a homeomorphism ψ from ∂(h∪B) to the boundary of a solid torus $H_{2}$ such that M is homeomorphic to the manifold pasted by h∪B and $H_{2}$ along ψ. We put $H_{1}=h\cup N(D_{1};W_{1})\cup N(D_{2};W_{1})$, $H_{3}=W_{2}$. Thus, $\left(H_{1},H_{2},H_{3}\right)$ is a type-(0,1,2) handlebody decomposition of M. By the construction, each meridian disc of $H_{2}$ and $H_{3}$ intersects the singular graph at least four and six times, respectively. Hence, the handlebody decomposition is unstabilized. ▪

### (b) Examples: the three-dimensional torus

We will show some examples of handlebody decompositions of the three-dimensional torus $T^{3}$.

**Figure 9.**
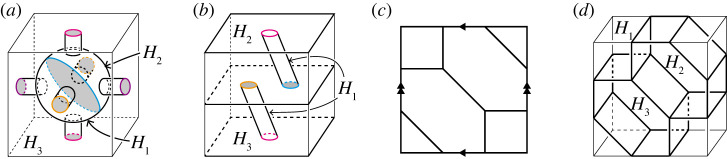
(*a*) A type-(0,0,3) handlebody decomposition of $T^{3}$. Coloured edges illustrate the singular graph, and greyed discs are all components of $F_{12}$ as in remark 2.2. (*b*) A type-(0,2,2) handlebody decomposition of $T^{3}$. (*c*) A decomposition of $T^{2}$ into three hexagons. (*d*) The hexagonal honeycomb decomposition of $T^{3}$. (Online version in colour.)

First, we consider handlebody decompositions consisting of one ball and two handlebodies. By proposition 4.2, $T^{3}$ admits a unstabilized type-(0,0,3) handlebody decomposition (figure 9*a*). Thus, for k≥0 and l≥3, $T^{3}$ admits a type-(0,k,l) handlebody decomposition by remark 3.2. Figure 9*b* illustrates a type-(0,2,2) handlebody decomposition of $T^{3}$. On the other hand, by theorem 4.1, $T^{3}$ admits neither type-(0,0,0), type-(0,0,1), nor type-(0,1,1) handlebody decompositions. In addition, by propositions 4.2 and 4.5, there is no type-(0,1,2) handlebody decomposition of $T^{3}$. Hence, any type-(0,2,2) handlebody decomposition of $T^{3}$ is unstabilized. In summary, we have the following proposition.

**Proposition 4.10.**

*Let* (k,l) *be a pair of non-negative integers with* k≤l. *The 3-dimensional torus* $T^{3}$ *admits a type*-(0,k,l) *handlebody decomposition if and only if the pair* (k,l) *is not in* {0,1}×{0,1,2}.

Theorem 4.1 guarantees that $T^{3}$ admits a type-(1,1,1) handlebody decomposition. Figure 9*c* shows a decomposition of $T^{2}$ into three hexagons. By taking the product with $S^{1}$, we have a decomposition of $T^{3}$ into three solid tori. We call this handlebody decomposition the *hexagonal honeycomb decomposition* (figure 9*d*). In general, a three-manifold admits a lot of handlebody decompositions of the same type. The next proposition asserts that the hexagonal honeycomb decomposition is the unique type-(1,1,1) handlebody decomposition of $T^{3}$ up to self-homeomorphism of $T^{3}$.

**Proposition 4.11.**

*For a simple and proper type*-(1,1,1) *handlebody decomposition of* $T^{3}$, *there exists a self-homeomorphism of* $T^{3}$ *that maps the partition of the decomposition to that of the hexagonal honeycomb decomposition*.

**Proof.**

Let $\left(H_{1},H_{2},H_{3}\right)$ be a simple and proper type-(1,1,1) handlebody decomposition of $T^{3}$. Let $F_{ij}$ denote a surface as in remark 2.2.

**Claim 4.12.**

For any 1≤i<j≤3, there is no disc component in $F_{ij}=H_{i}\cap H_{j}$.

**Proof of claim 4.12.**

Suppose there is a disc component D in some $F_{ij}$. Without loss of generality, we can assume that $D\subset F_{23}$. If ∂D is essential in $\partial H_{1}$, $H_{1}^{\prime }=H_{1}\cup N(D)$ is a punctured lens space. Since $T^{3}$ is prime, $H_{1}^{\prime }$ is a three-ball. Thus, a triple $\left(H_{1}^{\prime },\mathrm{cl}(H_{2}\setminus N(D)),\mathrm{cl}(H_{3}\setminus N(D))\right)$ gives a simple and proper type-(0,1,1) handlebody decomposition of $T^{3}$. However, from proposition 4.10, $T^{3}$ does not admit such a decomposition, which is a contradiction.

Suppose ∂D is inessential in $\partial H_{1}$. Then we can take a disc $D^{\prime }$ in $\partial H_{1}$ such that $\partial D^{\prime }=\partial D$. Put $S=D\cup D^{\prime }$. Since $T^{3}$ does not contain non-separating spheres, S is separating. Thus, $F_{23}$ consists of only the disc D. Hence, $H_{2}\cup H_{3}$ is a genus-2 handlebody. This is impossible because $\partial (H_{2}\cup H_{3})=\partial H_{1}$ is a torus. ▪

By claim 4.12 and [3, lemma 1], each component of $F_{ij}$ is an annulus. Suppose the core of an annulus of $F_{ij}$ is meridional in $H_{i}$. Then, C is longitudinal in $H_{j}$; otherwise, we can find a punctured lens space or a punctured $S^{2}\times S^{1}$ in $T^{3}$, which is a contradiction. Then, by removing the neighbourhood of a meridian disc in $H_{i}$ and attaching it to $H_{j}$, we have a decomposition of $T^{3}$ with two three-balls and a solid torus, i.e. a type-(0,0,1) decomposition, which contradicts proposition 4.10. Therefore, $H_{1}$, $H_{2}$ and $H_{3}$ are fibre tori of a Seifert fibration of $T^{3}$. As the Seifert fibre structure of $T^{3}$ is unique up to self-homeomorphism of $T^{3}$, we can assume that $H_{i}=D_{i}\times S^{1}$ (i=1,2,3), where $D_{1}$, $D_{2}$ and $D_{3}$ are discs in $T^{2}$ satisfying $D_{1}\cup D_{2}\cup D_{3}=T^{2}$. By the Euler characteristic, the intersection of two of the discs consists of precisely three arcs. Hence, this structure corresponds to the hexagonal honeycomb decomposition in $T^{3}$.

When it comes to the case of type-(2,2,2) handlebody decompositions, the uniqueness no longer holds, as we see in the following example.

**Example 4.13.**

**Figure 10.**
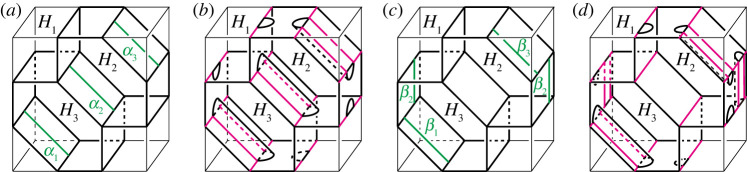
(*b*,*d*) A pair of different handlebody decompositions of $T^{3}$. The decomposition (*b*) (resp. (*d*)) is of type-(2,2,2) obtained from the hexagonal honeycomb decomposition by type-1 stabilizations along $\alpha_{1}$, $\alpha_{2}$ and $\alpha_{3}$ in (*a*) (resp. $\beta_{1}$, $\beta_{2}$ and $\beta_{3}$ in (*c*)). (Online version in colour.)

By performing type-1 stabilizations to the honeycomb decomposition three times, we have the two type-(2,2,2) handlebody decompositions shown in figure 10. In figure 10*a*, each $F_{ij}=H_{i}\cap H_{j}$ is homeomorphic to the disjoint union of a two-holed torus and a disc. On the other hand, in figure 10*b*, the surface $F_{23}$ is homeomorphic to the disjoint union of a one-holed torus and an annulus, and each $F_{1j}$ is homeomorphic to a three-holed sphere. Hence, they are different decompositions. ▪

## 5. Topological study of polycontinuous patterns

In this section, we will consider ‘polycontinuous patterns’, which are roughly three-periodic structures assembled by polymers. See, for example, [6,15] for studies on polycontinuous patterns. We will suggest a mathematical model of polycontinuous patterns (definition 5.8).

### (a) Polycontinuous patterns and net-like patterns

First, we define ‘net-like patterns’ that satisfy the essential properties of polycontinuous patterns.

**Definition 5.1.**

We denote by $T^{d}$ the d-dimensional torus. Let $\tilde{X}$ be a graph embedded in $R^{d}$ such that each component of $\tilde{X}$ is unbounded. If there exists a covering map $\pi :R^{d}\to T^{d}$ such that all covering transformations of π preserve $\tilde{X}$, then $\tilde{X}$ is called a *net*.

In this paper, we mainly discuss the case where d=3.

**Remark 5.2.**

In crystal chemistry (e.g. [16]), the term ‘net’ means a periodic, connected, simple, abstract graph. In this paper, we allow a net to be disconnected. Furthermore, all nets are embedded in Euclidean space.

**Definition 5.3.**

Let $\tilde{P}$ be a non-compact connected two-dimensional polyhedron embedded in $R^{3}$. The polyhedron $\tilde{P}$ is called a *net-like pattern* if there exist a covering map $\pi :R^{3}\to T^{3}$ and a net $\tilde{X}$ such that the following conditions hold:

(1) All covering transformations of π preserve both $\tilde{P}$ and $\tilde{X}$.
(2) The polyhedron $\tilde{P}$ divides $R^{3}$ into unbounded open components $V_{i}$ (i∈I), where I is a finite or countable set.
(3) There exists a strong deformation retraction of $R^{3}\setminus \tilde{P}$ onto $\tilde{X}$.

We call the pair $\left(\tilde{P},\pi \right)$ a *framed net-like pattern*, and π its *frame*. We say that a connected component of $R^{3}\setminus \tilde{P}$ (resp. $\tilde{X}$) is a *labyrinthine domain* (resp. *labyrinthine net*) of $\tilde{P}$.

A net-like pattern $\tilde{P}$ is said to be *proper* if there is no simple closed curve in $R^{3}$ that does not cross the singular graph and intersects a sector of $\tilde{P}$ transversely once. A net-like pattern $\tilde{P}$ is said to be *simple* if $\tilde{P}$ is a simple polyhedron.

More generally, we can define net-like patterns for any closed prime three-manifold with a (possibly non-Euclidean) crystallographic group and its covering space, but this paper will not deal with it.

**Remark 5.4.**

Consider two net-like patterns that satisfy the following conditions:

(1) They have the same labyrinthine net.
(2) They do not have a disc sector.
(3) The singular graphs of them have no vertices.

Then, they can be transformed to each other by a (possibly infinite) sequence of *IX-moves*, *XI-moves* and isotopies (see [17, theorem 3.1]).

By using [11,18], if two net-like patterns with the same labyrinthine net are simple, then the patterns can be transformed to each other by a (possibly infinite) sequence of 0-2 moves, 2-0 moves, 2-3 moves, 3-2 moves and isotopies.

The following two propositions state a relationship between (framed) net-like patterns and handlebody decompositions of $T^{3}$.

**Proposition 5.5.**

*Let* $\left(H_{1},H_{2},\ldots ,H_{n};P\right)$ *be a handlebody decomposition of* $T^{3}$, *and* $\tilde{P}$ *the preimage of* P *under the universal covering map* π *of* $T^{3}$. *Suppose that, for each* i, *the induced homomorphism* ${\left(\iota_{i}\right)}_{*}:\pi_{1}(H_{i})\to \pi_{1}(T^{3})$ *is not trivial, where* $\iota_{i}$ *is the inclusion map. Then the pair* $\left(\tilde{P},\pi \right)$ *is a framed net-like pattern. Furthermore, if* P *is simple (resp. proper), then the net-like pattern is also simple (resp. proper)*.

**Proof.**

Let $\left\{V_{j}^{i}\right\}$ be the connected components of the preimage of $H_{i}$ under π. Since the homomorphism ${\left(\iota_{i}\right)}_{*}$ is not trivial, each open component $V_{j}^{i}$ is unbounded. Each open handlebody $H_{i}$ contains a simple finite graph $X_{i}$ that is a strong deformation retract of $H_{i}$. Then the preimage, ${\tilde{X}}_{i}$, of $X_{i}$ under π is a net. Furthermore, each connected component of ${\tilde{X}}_{i}$ is a strong deformation retract of some $V_{j}^{i}$. Hence, $\left(\tilde{P},\pi \right)$ is a framed net-like pattern.

Since π is a local homeomorphism, if P is simple, then the preimage $\tilde{P}$ is also simple. Next, assume that the handlebody decomposition $\left(H_{1},H_{2},\ldots ,H_{n};P\right)$ of $T^{3}$ is proper, whereas the net-like pattern $\tilde{P}$ is *not* proper. Then, there exists a simple loop $\tilde{c}$ in $R^{3}$ that transversely intersects P at a single point only in a sector. Thus, there exists a simple loop in $T^{3}$ isotopic to $\pi (\tilde{c})$ that intersects a sector of P transversely once. Hence, the handlebody decomposition is not proper, which is a contradiction. Therefore, the net-like pattern $\;\tilde{P}$ is proper. ▪

**Proposition 5.6.**

*Let* $\left(\tilde{P},\pi \right)$ *be a framed net-like pattern*.

(1) *The image* $\pi (\tilde{P})$ *gives a handlebody decomposition of* $T^{3}$. *If* $\tilde{P}$ *is simple, then the handlebody decomposition is also simple*.
(2) *Let* ${\left\{V_{i}\right\}}_{i\in I}$ *be the set of labyrinthine domains of* $\tilde{P}$, *where* I *is a finite or countable set. Suppose that* $\tilde{P}$ *is proper. Suppose further that for any* $V_{i}$, $V_{j}$ *with* $V_{i}\neq V_{j}$, *where* $V_{i}$ *is the image of* $V_{j}$ *under some covering transformation*, $V_{i}$ *and* $V_{j}$ *are not adjacent to the same sector. Then the handlebody decomposition given by* $\pi (\tilde{P})$ *is proper*.

**Proof.**

Let Γ be the covering transformation group of π. Set $P=\pi (\tilde{P})$.

(1) Since $\tilde{P}$ is a connected two-dimensional polyhedron, its projection image P is also a connected two-dimensional polyhedron. Furthermore, if $\tilde{P}$ is simple, then P is also simple.

The complement $T^{3}\setminus P$ consists of finite open components $\left\{H_{j}\right\}$ because $T^{3}=R^{3}/\Gamma$ is compact and P is the underlying space of a locally finite complex. We show that each open component $H_{j}$ is an open handlebody. There exists a labyrinthine domain $V_{i}$ such that $H_{j}=\pi (V_{i})$. Furthermore, since $\tilde{P}$ is a net-like pattern, there exists a labyrinthine net ${\tilde{X}}_{i}$ such that ${\tilde{X}}_{i}$ is a strong deformation retract of $V_{i}$. Put $G=\pi ({\tilde{X}}_{i})$. Then, G is an embedding of a graph in $T^{3}$. So, the fundamental group $\pi_{1}(G)$ is free. Since $\pi {|}_{V_{i}}$ is a covering map, and ${\tilde{X}}_{i}$ is a strong deformation retract of $V_{i}$, the inclusion map $G\to H_{j}$ induces an isomorphism from $\pi_{1}(G)$ to $\pi_{1}(H_{j})$. Hence, $H_{j}$ is the interior of a handlebody because $\pi_{1}(H_{j})$ is free. Therefore, P gives a handlebody decomposition of $T^{3}$.

(2) We suppose that $\tilde{P}$ is proper and P is not proper. Then there exists a simple loop $c:[0,1]\to T^{3}$ such that it transversely intersects P at a single point only in a sector. Let $\tilde{c}$ be a lift of c. Since $\tilde{P}$ is proper, $\tilde{c}$ is an arc (not a loop) whose initial point v and terminal point w are contained in different labyrinthine domains $V_{i}$ and $V_{j}$, respectively, of $R^{3}\setminus \tilde{P}$. Note that $V_{i}$ and $V_{j}$ are adjacent. This is impossible because there exists a covering transformation that takes v to w, so $V_{i}$ to $V_{j}$. ▪

**Example 5.7.**

**Figure 11.**
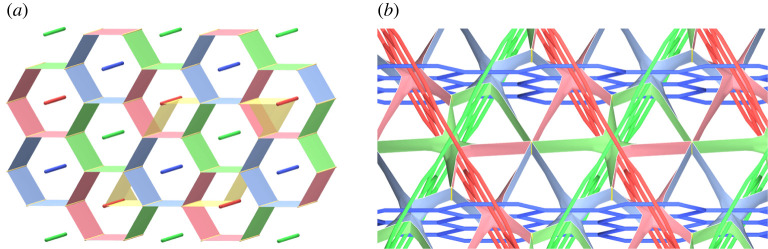
(*a*) The hexagonal honeycomb pattern and (*b*) a non-simple net-like pattern. (Online version in colour.)

Figure 11*a* illustrates a simple proper net-like pattern that comes from the hexagonal honeycomb tessellation of $R^{2}$. A yellow polygon illustrates a fundamental domain of its frame. We call the pattern the *hexagonal honeycomb pattern*. By proposition 5.6, the hexagonal honeycomb pattern with the frame induces the hexagonal honeycomb decomposition (see figure 9*d*). Note that a tessellation of $R^{2}$ induces a net-like pattern in general. The meanings of colours except yellow in figure 11*a* will be explained in definition 5.10.

We now suggest a strict mathematical definition of polycontinuous patterns.

**Definition 5.8.**

We say that a net-like pattern$\;\tilde{P}$ is an *n-continuous pattern* (or a *polycontinuous pattern*) if the following conditions hold:

(i) $\tilde{P}$ has precisely n labyrinthine domains.
(ii) $\tilde{P}$ is proper.
(iii) Any sector of $\tilde{P}$ is not a disc.

Note that for any positive integer n, there exists an n-continuous pattern. In the remainder, we call a two-continuous (resp. three-continuous) pattern a *bicontinuous* (resp. *tricontinuous*) pattern, according to the conventions of soft materials [6,15].

The following corollary is a polycontinuous pattern version of proposition 5.5.

**Corollary 5.9.**

*Let* $\left(H_{1},\ldots ,H_{n};P\right)$ *be a proper handlebody decomposition of* $T^{3}$, *and* $\tilde{P}$ *the preimage of* P *under the universal covering map of* $T^{3}$. *Suppose that the following two conditions hold*:

(1) *For each* i, *we have* ${\left(\iota_{i}\right)}_{*}(\pi_{1}(H_{i}))≅Z⊕Z⊕Z$.
(2) *Any sector of* P *is not a disc*.

*Then*, $\tilde{P}$ *is a polycontinuous pattern. In particular, if* ${\left(\iota_{i}\right)}_{*}(\pi_{1}(H_{i}))=\pi_{1}(T^{3})$ *for each* i, *then* $\tilde{P}$ *is an* n-*continuous pattern. Furthermore, if* P *is simple (resp. proper), then* $\tilde{P}$ *is also simple (resp. proper)*.

### (b) Colourings of patterns

In this subsection, we will define colourings of net-like patterns. Each labyrinthine domain of a net-like pattern is a mathematical model of polymers assembled in one kind of block. In materials science, one kind of block may form many domains of a net-like pattern in general. To describe such a situation, we introduce ‘colours’ of net-like patterns, of which each colour corresponds to one kind of block of polymers.

**Definition 5.10.**

Let $\tilde{P}$ be a net-like pattern. Set $X_{n}=\{1,2,\ldots ,n\}$. Let $\tilde{V}$ denote the set of all labyrinthine domains of $\tilde{P}$. A surjection $\varphi :\tilde{V}\to X_{n}$ is called an *n-colouring of $\tilde{P}$* if there exists a frame π of $\tilde{P}$ such that the following conditions hold:

(1) For each covering transformation t of π and for any $V\in \tilde{V}$, we have φ(V)=φ∘t(V).
(2) Two sides of a local part of each sector have different colours. Namely, for each point p in a sector C, the two labyrinthine domains, V and $V^{\prime }$, that have non-trivial intersection with N(p) satisfy $\varphi (V)\neq \varphi (V^{\prime })$.

The image φ(V) is called *the colour of V*. The frame π of $\tilde{P}$ is said to be *compatible with the colouring φ*. A net-like pattern together with a fixed (n-) colouring is called an *(n-)coloured* net-like pattern (figure 11*b*). We say that two coloured net-like patterns, $\tilde{P}$ and $\tilde{Q}$, are *equivalent* if there exists an ambient isotopy of $R^{3}$ that moves $\tilde{P}$ to $\tilde{Q}$, and each pair of corresponding labyrinthine domains has the same colour after permuting the colours. If a surjection φ satisfies only the condition (1), then we call φ a *non-effective n-colouring*, and $\tilde{P}$ is said to be *non-effectively n-coloured*.

Let $\tilde{P}$ be a (possibly non-effectively) n-coloured net-like pattern with a colouring $\varphi :\tilde{V}\to X_{n}$, where $X_{n}=\{1,2,\ldots ,n\}$, and π a frame of $\tilde{P}$ compatible with φ. By proposition 5.6, the image $\pi (\tilde{P})$ gives a handlebody decomposition of $T^{3}$. Denote by V the set of all handlebodies of the decomposition. Then we say that $\left(\tilde{P},\pi \right)$ is *of type $\left(g_{1},\ldots ,g_{n}\right)$*, where $g_{i}=[g_{i_{1}},\ldots ,g_{i_{k}}]$ is a sequence of the genera of the handlebodies in V coloured by $i\in X_{n}$. (For simplicity, if the length of $g_{i}$ is equal to 1, then we put $g_{i}=g_{i_{1}}$.)

Note that, as opposed to colourings of graphs on surfaces, for any integers m, n with n≥2 and m<n, there is an n-coloured framed net-like pattern that does not admit m-colouring.

**Remark 5.11.**

If a net-like pattern admits a colouring, then it is necessarily proper.

In fact, a coloured net-like pattern $\;\tilde{P}$ with its frame π compatible with the colouring satisfies the condition that $\pi (\tilde{P})$ induces a proper handlebody decomposition of $T^{3}$ since any two labyrinthine domains sharing a sector have different colours (see proposition 5.6). Hence, the following holds.

**Corollary 5.12.**

*Let* $\left(\tilde{P},\pi \right)$ *be a framed net-like pattern and let* $g_{i}=[g_{1}^{\left(i\right)},\ldots ,g_{k_{i}}^{\left(i\right)}]$ *be a sequence of positive integers for* i∈{1,…,n}. *Suppose that* $\left(\tilde{P},\pi \right)$ *admits an* n-*colouring and is of type* $\left(g_{1},\ldots ,g_{n}\right)$. *Then* $\pi (\tilde{P})$ *gives a proper type*-$\left(g_{1}^{\left(1\right)},\ldots ,g_{k_{1}}^{\left(1\right)},\ldots ,g_{1}^{\left(n\right)},\ldots ,g_{k_{n}}^{\left(n\right)}\right)$ *handlebody decomposition* $\left(H_{1}^{\left(1\right)},\ldots ,H_{k_{1}}^{\left(1\right)},\ldots ,H_{1}^{\left(n\right)},\ldots ,H_{k_{n}}^{\left(n\right)}\right)$ *such that* $H_{j_{1}}^{\left(i\right)}\cap H_{j_{2}}^{\left(i\right)}=\emptyset$ *for* $j_{1},j_{2}\in \{1,\ldots ,k_{i}\}$.

The converse of the above corollary is clear by proposition 5.5.

**Corollary 5.13.**

*Let* $\left(H_{1}^{\left(1\right)},\ldots ,H_{k_{1}}^{\left(1\right)},\ldots ,H_{1}^{\left(n\right)},\ldots ,H_{k_{n}}^{\left(n\right)};P\right)$ *be a proper type*-$(g_{1}^{\left(1\right)},$ …, $g_{k_{1}}^{\left(1\right)},$ …, $g_{1}^{\left(n\right)},$ …, $g_{k_{n}}^{\left(n\right)})$ *handlebody decomposition of* $T^{3}$ *and let* π *be the universal covering map* $R^{3}\to T^{3}$. *We assume that* $H_{j_{1}}^{\left(i\right)}\cap H_{j_{2}}^{\left(i\right)}=\emptyset$ *for* $j_{1},j_{2}\in \{1,\ldots ,k_{i}\}$. *We further assume that, for each handlebody* $H_{j}^{\left(i\right)}$, *the induced homomorphism* ${\left(\iota_{j}^{\left(i\right)}\right)}_{*}:\pi_{1}(H_{j}^{\left(i\right)})\to \pi_{1}(T^{3})$ *is not trivial, where* $\iota_{j}^{\left(i\right)}$ *is the inclusion map. Then* $\left(\pi^{-1}(P),\pi \right)$ *is a coloured net-like pattern of type* $\left(g_{1},\ldots ,g_{n}\right)$, *where* $g_{i}=[g_{1}^{\left(i\right)},\ldots ,g_{k_{i}}^{\left(i\right)}]$.

### (c) A sufficient condition for the equivalence of patterns

Corollaries 5.12 and 5.13 say there is a nice relationship between coloured net-like patterns and proper handlebody decompositions. This subsection gives a sufficient condition for two coloured net-like patterns to be equivalent.

To this end, we first consider adjusting a framed net-like pattern to another frame. Let $\left(\tilde{P},\pi \right)$ be a framed net-like pattern, and let ρ be a covering map $R^{3}\to T^{3}$. Since the two covering maps are equivalent, there exists a self-homeomorphism f of $R^{3}$ such that π=ρ∘f. If f is orientation-preserving, we say that π and ρ *have the same orientation*. Otherwise, we say that π and ρ *have different orientations*.

If π and ρ have the same orientation, then $\tilde{P}$ is isotopic to $f(\tilde{P})$, and $\left(f(\tilde{P}),\rho \right)$ is a framed net-like pattern. Consider the case that π and ρ have different orientations. Let r be an orientation-reversing self-homeomorphism of $T^{3}$. Then, $\pi^{\prime }:=r\circ \pi$ is also a covering map $R^{3}\to T^{3}$. Hence, there exists an orientation-preserving homeomorphism $g:R^{3}\to R^{3}$ such that $\pi^{\prime }=\rho \circ g$. So, $\tilde{P}$ is isotopic to $g(\tilde{P})$, and we have $r(\pi (\tilde{P}))=\rho (g(\tilde{P}))$. Furthermore, $\left(g(\tilde{P}),\rho \right)$ is a framed net-like pattern because each covering transformation of $\pi^{\prime }$ is also that of π.

To summarize, we obtain the following lemma.

**Lemma 5.14.**

*Let* $\left(\tilde{P},\pi \right)$ *be a framed net-like pattern, and let* ρ *be a covering map* $R^{3}\to T^{3}$. *Then, there exists a net-like pattern*$\;\tilde{Q}$ *such that the following three conditions hold*:

(1) *The covering map* ρ *is a frame of* $\tilde{Q}$.
(2) *The pattern* $\tilde{P}$ *is isotopic to* $\tilde{Q}$.
(3) *Either* $\pi (\tilde{P})=\rho (\tilde{Q})$ *or there exists an orientation-reversing self-homeomorphism* r *of* $T^{3}$ *with* $r(\pi (\tilde{P}))=\rho (\tilde{Q})$.

*In particular, if* $\tilde{P}$ *is* (*non-effectively*) n-*coloured, then so is* $\tilde{Q}$. *Furthermore*, $\tilde{P}$ *and* $\tilde{Q}$ *have the same type*.

By lemma 5.14, we can assume that any two net-like patterns have the same frame. Proposition 5.6 says the two framed net-like patterns induce two handlebody decompositions of $T^{3}$, respectively. If the two decompositions are isotopic, then the two patterns are also isotopic. In fact, we can say more as follows.

**Lemma 5.15.**

*Let* $\left(\tilde{P},\pi \right)$ *and* $\left(\tilde{Q},\pi \right)$ *be framed net-like patterns. Suppose that there exists an orientation-preserving self-homeomorphism* f *of* $T^{3}$ *that maps* $\pi (\tilde{P})$ *to* $\pi (\tilde{Q})$. *Then*, $\tilde{P}$ *is isotopic to* $\tilde{Q}$.

**Proof.**

By the assumption, we have an orientation-preserving self-homeomorphism f of $T^{3}$ that maps $\pi (\tilde{P})$ to $\pi (\tilde{Q})$. Let $\tilde{f}$ be the unique lift of f∘π. Then $\tilde{f}$ is a homeomorphism of $R^{3}$, and we have $\tilde{f}(\tilde{P})=\tilde{Q}$. Therefore, $\tilde{P}$ and $\tilde{Q}$ are isotopic. ▪

By the above lemmas, we have the following proposition.

**Proposition 5.16.**

*Let* $\left(\tilde{P},\pi \right)$ *and* $\left(\tilde{Q},\rho \right)$ *be* n-*coloured framed net-like patterns. We assume that* $\pi (\tilde{P})$ *and* $\rho (\tilde{Q})$ *are homeomorphic under an orientation-preserving or orientation-reversing self-homeomorphism* f *of* $T^{3}$ *according to whether the covering maps* π *and* ρ *have the same orientation or different orientations. Suppose that any two corresponding handlebodies under* f *are the images of labyrinthine domains with the same colour (after permuting the colours). Then* $\left(\tilde{P},\pi \right)$ *and* $\left(\tilde{Q},\rho \right)$ *are equivalent*.

## 6. Stabilizations on net-like patterns

In this section, we will discuss (de)stabilizations of net-like patterns and introduce some examples.

### (a) Stabilization theorem for net-like patterns

Section 3 showed an analogue of the Reidemeister–Singer theorem for handlebody decompositions (theorem 3.5). This subsection shows a net-like pattern version of the theorem. To do so, we define (de)stabilizations of net-like patterns. First, we will use an example to explain how to define it.

**Figure 12.**
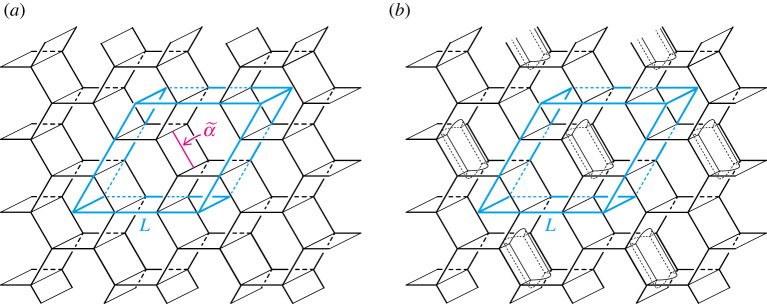
(*a*) The hexagonal honeycomb pattern introduced in example 5.7. The parallelepiped L illustrates a fundamental domain of the pattern. (*b*) The net-like pattern obtained by performing a type-1 stabilization on the honeycomb pattern along the properly embedded lifted arc $\tilde{\alpha }$. (Online version in colour.)

In example 5.7, we introduced the hexagonal honeycomb pattern that is a three-coloured framed net-like pattern of type (1,1,1). We consider a type-1 stabilization on the pattern. Let $\tilde{\alpha }$ be a properly embedded arc in a sector of the pattern (figure 12*a*). We assume that $\tilde{\alpha }$ is *lifted by π*, i.e. the restriction of π to $\tilde{\alpha }$ is injective, where π is the frame of the pattern. By corollary 5.12, $\pi (\tilde{P})$ gives a simple proper handlebody decomposition. As noted in example 5.7, $P:=\pi (\tilde{P})$ is a simple proper type-(1,1,1) handlebody decomposition (figure 9*d*). Since $\tilde{\alpha }$ is lifted and contained in a sector, the image $\alpha :=\pi (\tilde{\alpha })$ is a properly embedded arc in a sector of P. Furthermore, $\tilde{\alpha }$ connects two labyrinthine domains mapped to the same handlebody by π. So, the arc α connects the same handlebody. Thus, we can perform a type-1 stabilization along α. Hence, a type-(1,1,2) handlebody decomposition $P^{\prime }$ is obtained by performing a type-1 stabilization along α. By corollary 5.13, the preimage of $P^{\prime }$ under π gives a three-coloured framed net-like pattern $\;{\tilde{P}}^{\prime }$ of type (1,1,2) (figure 12*b*). Hence, we obtain the new net-like pattern ${\tilde{P}}^{\prime }$ from $\tilde{P}$. We will call such an operation a *type-1 stabilization for net-like patterns*.

Based on the above example, we give a strict definition of stabilizations for patterns as follows.

**Definition 6.1.**

Let $\left(\tilde{P},\pi \right)$ be a simple, n-coloured, framed net-like pattern of type $\left(g_{1},\ldots ,g_{n}\right)$, where $g_{i}$ is a sequence of positive integers $\left[g_{1}^{\left(i\right)},\ldots ,g_{m_{i}}^{\left(i\right)}\right]$ for 1≤i≤n. Put $P=\pi (\tilde{P})$. By corollary 5.12, P gives a simple proper handlebody decomposition $H=(H_{1}^{\left(1\right)},\ldots ,H_{m_{1}}^{\left(1\right)},H_{1}^{\left(2\right)},\ldots ,H_{m_{2}}^{\left(2\right)},\ldots ,H_{1}^{\left(n\right)},\ldots ,H_{m_{n}}^{\left(n\right)};P)$ of $T^{3}$ such that $H_{j}^{\left(i\right)}$ is a genus-$g_{j}^{\left(i\right)}$ handlebody coloured by i. Let U, $U^{\prime }$, V, $V^{\prime }$ and W be labyrinthine domains. We assume that, for each pair of the labyrinthine domains except for $\left(U,U^{\prime }\right)$, $\left(V,V^{\prime }\right)$ and (U,W), the two domains are different and share a sector. (There is a possibility that $U=U^{\prime }$ or $V=V^{\prime }$.) We further assume that $\pi (U)=\pi (U^{\prime })=H_{j_{U}}^{\left(i_{U}\right)}$, $\pi (V)=\pi (V^{\prime })=H_{j_{V}}^{\left(i_{V}\right)}$ and $\pi (W)=H_{j_{W}}^{\left(i_{W}\right)}$. Here, $H_{j_{U}}^{\left(i_{U}\right)}$, $H_{j_{V}}^{\left(i_{V}\right)}$ and $H_{j_{W}}^{\left(i_{W}\right)}$ are distinct handlebodies, and $i_{U}\neq i_{V}$, $i_{V}\neq i_{W}$ and $i_{W}\neq i_{U}$.

Depending on the type of stabilization, we take an arc $\tilde{\alpha }$ as follows.

(type-0) The arc $\tilde{\alpha }$ is a properly embedded lifted arc in V that connects U and $U^{\prime }$. We assume that a lifted disc in V contains $\tilde{\alpha }$ as a part of its boundary, and the other part is contained in ∂V.
(type-1) The arc $\tilde{\alpha }$ is a properly embedded lifted arc in a sector of $\tilde{P}$ that connects V and $V^{\prime }$.
(type-2) The arc $\tilde{\alpha }$ is a properly embedded lifted arc in V that connects U and W. We assume that a lifted disc in V contains $\tilde{\alpha }$ as a part of its boundary, and the other part is contained in ∂V.

Then, we can obtain a new handlebody decomposition $H^{\prime }$ performed by a suitable stabilization on H along $\pi (\tilde{\alpha })$. We can see by corollary 5.13 that the preimage of the partition of $H^{\prime }$ is a simple, coloured, framed net-like pattern of type $\left(g_{1}^{\prime },\ldots ,g_{n}^{\prime }\right)$. Here, each $g_{i}^{\prime }$ is equal to $g_{i}$ except for the following sequences:

(type-0) $g_{i_{U}}^{\prime }=[g_{1}^{\left(i_{U}\right)},\ldots ,g_{j_{U}}^{\left(i_{U}\right)}+1,\ldots ,g_{m_{i_{U}}}^{\left(i_{U}\right)}],\;g_{i_{V}}^{\prime }=[g_{1}^{\left(i_{V}\right)},\ldots ,g_{j_{V}}^{\left(i_{V}\right)}+1,\ldots ,g_{m_{i_{V}}}^{\left(i_{V}\right)}].$
(type-1 and type-2) $g_{i_{V}}^{\prime }=[g_{1}^{\left(i_{V}\right)},\ldots ,g_{j_{V}}^{\left(i_{V}\right)}+1,\ldots ,g_{m_{i_{V}}}^{\left(i_{V}\right)}].$

We call this operation a *type-k stabilization (along $\tilde{\alpha }$ with respect to π)* and its inverse operation a *type-k destabilization* for each k. In electronic supplementary material, we discuss sufficient conditions for performing a destabilization.

Note that the result of a (de)stabilization of a polycontinuous pattern is not necessarily a polycontinuous pattern. Further note that in a type-2 stabilization along an arc for net-like patterns, even if the arc connects different labyrinthine domains, we cannot perform the operation if they are the same colour.

Definition 3.4 introduced some operations for handlebody decompositions called *moves*. We next consider a net-like pattern version of them. Of course, we can perform the original operations on simple coloured net-like patterns, but they generally lose periodicity after performing them. Thus, we give adapted ‘moves’ to net-like patterns in a similar way to the stabilizations.

**Definition 6.2.**

Let $\left(\tilde{P},\pi \right)$ be a simple, n-coloured, framed net-like pattern. Take a properly embedded lifted arc $\tilde{\alpha }$ in a sector (resp. an edge $\tilde{\alpha }$ of the singular graph of $\tilde{P}$) so that it connects labyrinthine domains V and $V^{\prime }$ of different colours. By corollary 5.12, $P:=\pi (\tilde{P})$ gives a simple proper handlebody decomposition H. Then, we can obtain a new handlebody decomposition $H^{\prime }$ performed by a 0-2 (resp. 2-3) move on P along $\pi (\tilde{\alpha })$. By corollary 5.13 the preimage of the partition of $H^{\prime }$ is a simple, coloured, framed net-like pattern. We call such an operation a *0-2 (resp. 2-3) move (along $\tilde{\alpha }$ with respect to π)* and its inverse operation a *2-0 (resp. 3-2) move*.

Note that, similar to type-2 stabilizations of net-like patterns, even if we can perform a move on a handlebody decomposition corresponding to a pattern, it does not necessarily mean that we can perform the corresponding move on the pattern.

An analogue of the Reidemeister–Singer theorem for net-like patterns is as follows.

**Corollary 6.3.**

*Let* $\left(\tilde{P},\pi \right)$ *and* $\left(\tilde{Q},\rho \right)$ *be simple*, n-*coloured, framed net-like patterns of type* $\left(g_{1},\ldots ,g_{n}\right)$ *and* $\left(g_{1}^{\prime },\ldots ,g_{n}^{\prime }\right)$, *respectively, where* $g_{i}$ *and* $g_{i}^{\prime }$ *are positive integers. Then* $\tilde{P}$ *and* $\tilde{Q}$ *are equivalent after applying 0-2, 2-0 and 2-3 moves, and type*-0 *and type*-1 *stabilizations finitely many times*.

**Proof.**

We assume that π and ρ have the same orientation. The proof of the other case is similar. By corollary 5.12, the images $P:=\pi (\tilde{P})$ and $Q:=\rho (\tilde{Q})$ give type-$\left(g_{1},\ldots ,g_{n}\right)$ and type-$\left(g_{1}^{\prime },\ldots ,g_{n}^{\prime }\right)$ simple proper handlebody decompositions of $T^{3}$, respectively. Hence, by theorem 3.5, there exists a simple proper handlebody decomposition such that $\pi (\tilde{P})$ and $\rho (\tilde{Q})$ are isotopic to the partition R of the decomposition after applying 0-2, 2-0 and 2-3 moves, and type-0 and type-1 stabilizations to them finitely many times. By corollary 5.13, $\tilde{R}:=\pi^{-1}(R)$ is a simple n-coloured net-like pattern. Therefore, by proposition 5.16, each of $\tilde{P}$ and $\tilde{Q}$ is equivalent to $\tilde{R}$ after applying 0-2, 2-0 and 2-3 moves, and type-0 and type-1 stabilizations finitely many times. ▪

In the above corollary, we assume that, for each colour, all labyrinthine domains coloured by it are mapped to the same handlebody because moves performed in the proof of theorem 3.5 generally do not preserve the colouring. On the concept of colourings, we can regard single-coloured domains as composed of the same kind of blocks, so connecting these parts is a natural idea.

**Definition 6.4.**

Let $\left(\tilde{P},\pi \right)$ be a simple, n-coloured, framed net-like pattern. Take a properly embedded lifted arc $\tilde{\alpha }$ in a sector so that it connects labyrinthine domains, V and $V^{\prime }$, of the same colour. We assume that H:=π(V) and $H^{\prime }:=\pi (V^{\prime })$ are different handlebodies of the simple proper handlebody decomposition induced by $P:=\pi (\tilde{P})$. By performing a 0-2 move on P along $\pi (\tilde{\alpha })$, the modified H and $H^{\prime }$ are intersected, and by corollary 5.12, their intersection consists of only the disc created by the operation. So, $H^{\prime \prime }:=H\cup H^{\prime }$ is also a handlebody. Hence, we have a new handlebody decomposition by replacing H and $H^{\prime }$ with $H^{\prime \prime }$. By corollary 5.13, the decomposition induces a new simple, coloured, framed net-like pattern $\;({\tilde{P}}^{\prime },\pi )$. We call such an operation a *domain-connection (along $\tilde{\alpha }$ with respect to π)* and its inverse operation a *domain-disconnection*.

**Remark 6.5.**

We can obtain the type of $\left({\tilde{P}}^{\prime },\pi \right)$ in definition 6.4 as follows. Let $g_{i}$ be the sequence of positive integers in the type of $\left(\tilde{P},\pi \right)$ corresponding to the colour of the labyrinthine domains V and $V^{\prime }$. We remove the integers corresponding to V and $V^{\prime }$ from $g_{i}$ and append their sum. Then, we denote a new sequence by $g_{i}^{\prime }$. By replacing $g_{i}$ with $g_{i}^{\prime }$, we obtain the type of $\left({\tilde{P}}^{\prime },\pi \right)$.

By applying the following to a coloured net-like pattern, it satisfies the assumption of corollary 6.3.

**Lemma 6.6.**

*Let* $\left(\tilde{P},\pi \right)$ *be a simple*, n-*coloured, framed net-like pattern of type* $\left(g_{1},\ldots ,g_{n}\right)$, *where* $g_{i}$ *is a sequence of positive integers* $\left[g_{1}^{\left(i\right)},\ldots ,g_{m_{i}}^{\left(i\right)}\right]$ *for* 1≤i≤n. *Set* ${\hat{g}}_{i}=\sum_{k=1}^{m_{i}}g_{k}^{\left(i\right)}$. *Then, we have a simple*, n-*coloured, framed net-like pattern of type* $\left({\hat{g}}_{1},\ldots ,{\hat{g}}_{n}\right)$ *by applying 0-2 moves and domain-connections with respect to* π *finitely many times to* $\tilde{P}$.

**Proof.**

Since $\tilde{P}$ is connected, for each colour i, there exist labyrinthine domains, V and $V^{\prime }$, with colour i and an embedded lifted arc $\tilde{\delta }$ joining V and $V^{\prime }$ in $\tilde{P}$ such that the following hold:

(1) The images π(V) and $\pi (V^{\prime })$ are distinct handlebodies.
(2) The arc $\tilde{\delta }$ does not cross any labyrinthine domains with colour i.
(3) The arc $\tilde{\delta }$ intersects the singular graph of $\tilde{P}$ transversely.

Then, by cutting $\tilde{\delta }$ at its intersection with the singular graph, we obtain the sequence of sub-arcs ${\tilde{\delta }}_{1},\ldots ,{\tilde{\delta }}_{k}$. Thus, we can perform 0-2 moves along ${\tilde{\delta }}_{1}$, …, ${\tilde{\delta }}_{k-1}$, and we can finally apply domain-connection along ${\tilde{\delta }}_{k}$. By repeating the above process, all labyrinthine domains with colour i are joined. Then, the type corresponding to colour i is ${\hat{g}}_{i}$ by remark 6.5. Therefore, we have a net-like pattern of type $\left({\hat{g}}_{1},\ldots ,{\hat{g}}_{n}\right)$. ▪

### (b) Microphase separation of a block copolymer melt

One motivation for this research comes from materials science. We are interested in the characterization of *polycontinuous patterns* that appear as microphase separation of a block copolymer melt [6,7].

**Figure 13.**
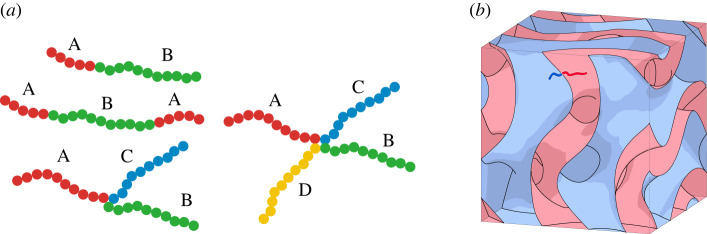
(*a*) A red dot indicates the monomer A, green monomer B, blue monomer C and yellow monomer D. Left: AB diblock copolymer, ABA triblock copolymer and ABC star-shaped block copolymer. Right: ABCD star-shaped block copolymer. (*b*) A double gyroid that is a famous bicontinuous pattern. (Online version in colour.)

In this subsection, we discuss block copolymers and phase separation of a block copolymer melt. One reference of this subject is [19]. A *polymer* is a molecule of high molecular weight created by chemically coupling large numbers of small reactive molecules, called *monomers*. If a polymer is made of one type of monomer, it is called a *homopolymer*. A polymer containing two or more chemically distinct monomers is referred to as a *copolymer*. A *block copolymer* is an important type of copolymer, in which monomers of a given type form polymerized sequences called *blocks*. If a block copolymer contains two (respectively three) blocks, it is called a *diblock* (resp. *triblock*) copolymer. If a linear diblock copolymer is made of blocks of monomers A and B, it is called an *AB diblock copolymer*. An *ABA triblock copolymer* is a linear triblock copolymer consisting of a sequence of a block of monomer A, a block of monomer B, and a block of monomer A. See figure 13*a*. SBS (styrene–butadiene–styrene) triblock and SIS (styrene–isoprene–styrene) triblock copolymers are examples of linear triblock copolymers. Polymers with more complex architecture have been synthesized. For example, a *star polymer* has one branched point connecting several linear polymers. An *ABC triblock-arm star-shaped molecule* (*3-star polymer*) as in figure 13*a* is an example of triblock copolymer with a star architecture, where A, B and C blocks are mutually immiscible.

A *block copolymer melt* is a solvent-free viscoelastic liquid composed of block copolymers. Due to the chemical distinction of monomers, we can observe phase separation in a block copolymer melt. A *domain* of phase separation consists of monomers of one type. *Microphase separation* of a block copolymer melt is phase separation with domains of the mesoscopic size scale. Sphere, cylinder, bicontinuous and lamellar structures appear as microphase separation of AB diblock or ABA triblock copolymers [19,20].

An example of *bicontinuous patterns* is the Gyroid surface. In materials science, in the bicontinuous pattern of an AB diblock copolymer melt, the domain of the A monomer is the neighbourhood of the partition surface, and that of the B monomer forms two labyrinths (figure 13*b*). A *tricontinuous pattern* is a mathematical model of microphase separation of an ABC star-shaped block copolymer melt. The branch line of a tricontinuous pattern consists of the connection points of the A, B and C blocks in the block copolymers [6]. See [21–23] for studies on geometric phases of star polymer melts. Note that a sector of a tricontinuous pattern is the interface of two domains.

Next, we discuss a mathematical model of microphase separation with four phases. Let A, B, C and D be four chemically distinct monomers. We consider the polycontinuous pattern of melts of four types of three-star block copolymers of ABC, ABD, ACD and BCD. In this case, four different branched lines appear. The interface of domains and these branched lines form a simple polyhedron. The vertex of the simple polyhedron of the polycontinuous pattern corresponds to the point where four domains A, B, C and D meet. The four edges corresponding to the connecting points of the ABC, ABD, ACD and BCD triblock star polymers are placed around a vertex. Also, ABCD four-star polymers are synthesized [24,25], and their morphologies have been discussed in [26,27]. The joining point of four blocks of the block copolymer corresponds to a vertex of the simple polycontinuous pattern.

We want to analyse the property of materials with this structure via a topological study of these polycontinuous patterns. We hope the characterization and the classification of polycontinuous patterns will lead to the design of polymeric materials with the desired properties.

As an application of corollary 6.3, we can discuss the relation between two microphase-separated structures of the same type. Here, we discuss the polymer science implications of stabilization and destabilization operation of patterns.

**Observation 6.7.**

The type-0 destabilization for a bicontinuous pattern can be considered as the model of the cancelling of an unstable local one-handle structure of the pattern of the microphase separation. The type-1 destabilization (resp. stabilization) for a polycontinuous pattern can be considered as the model of the separation (resp. amalgamation) of the domains during the uniaxial elongation of polymeric materials.

### (c) Example: a 3srs pattern

A 3srs pattern is an example of a tricontinuous pattern. In this subsection, we will show the pattern can be destabilized to the hexagonal honeycomb pattern.

**Figure 14.**
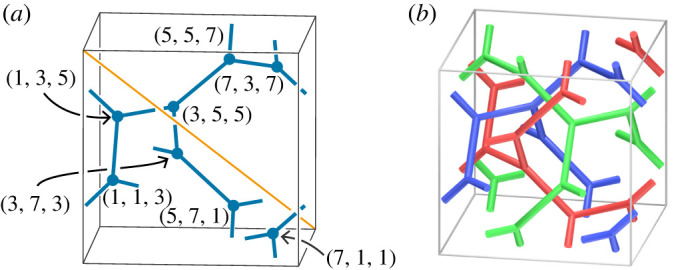
(*a*) An srs net. The orange line l passes through the points (0,0,8) and (8,8,0). Note that this net is topologically the same as the *srs-b* net (see [30]). (*b*) A 3srs net. The 2π/3 rotation around l preserves the net. (Online version in colour.)

First, we introduce a 3srs net. An *srs* net is a 3-periodic ‘minimal’ net in $R^{3}$ (see [28] and figure 14*a*). Figure 14*a* illustrates an srs net with a cubical fundamental domain, of which the length of each edge is 8. The net is an infinite trivalent graph, and the *space group* of it is $I4_{1}32$ (see [15,29]). Note that a 2π/3 rotation around the cube diagonal (shown in figure 14*a*) generates an action of order 3 and preserves the cube. A *3srs* net is the union of the images of the srs net under the action (figure 14*b*).

**Figure 15.**
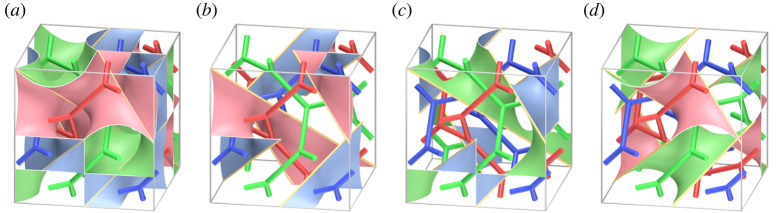
(*a*) The tricontinuous pattern corresponding to the 3srs net with a cubical fundamental domain. (*b*–*d*) Surfaces with boundary, each of which is shared by exactly two labyrinthine domains. (Online version in colour.)

Figure 15*a* illustrates a branched surface in $R^{3}$ with a cubical fundamental domain. The branched surface is the union of precisely three surfaces with the boundary (figure 15*b*–*d*). It is clear that the branched surface is a simple three-coloured tricontinuous pattern, and each component of the 3srs net is a labyrinthine net of the pattern. We call the tricontinuous pattern the *3srs pattern*. The 3srs pattern is of type (5,5,5) as illustrated in figures 14 and 15.

**Theorem 6.8.**

*The 3srs pattern can be destabilized to the hexagonal honeycomb pattern, i.e. the 3srs pattern can be obtained from the hexagonal honeycomb pattern by a finite sequence of type-1 stabilizations*.

**Proof.**

**Figure 16.**
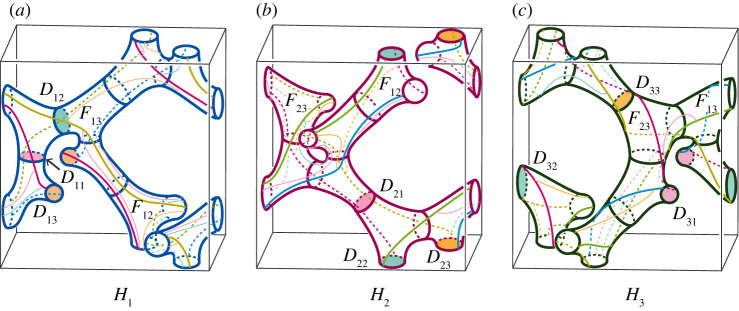
A handlebody decomposition of $T^{3}$ induced by the 3srs pattern. The ‘cores’ of handlebodies are the quotient of the 3srs net. The bold curves on the boundaries of handlebodies make up the singular graph. Each $F_{ij}$ denotes a surface defined in remark 2.2.

Let $\tilde{P}$ be the 3srs pattern, and π its frame obtained from a cubic fundamental domain as shown in figure 15. Put $P=\pi (\tilde{P})$. Figure 16 shows a simple proper type-(5,5,5) handlebody decomposition $\left(H_{1},H_{2},H_{3};P\right)$ of $T^{3}$ induced by $\tilde{P}$. We denote by $F_{12}$, $F_{13}$ and $F_{23}$ surfaces with boundary as in remark 2.2. By definition 6.1, if we destabilize the decomposition to the hexagonal honeycomb decomposition by performing a finite sequence of type-1 destabilizations, then we can also destabilize $\tilde{P}$ to the hexagonal honeycomb pattern by corresponding destabilizations.

**Figure 17.**
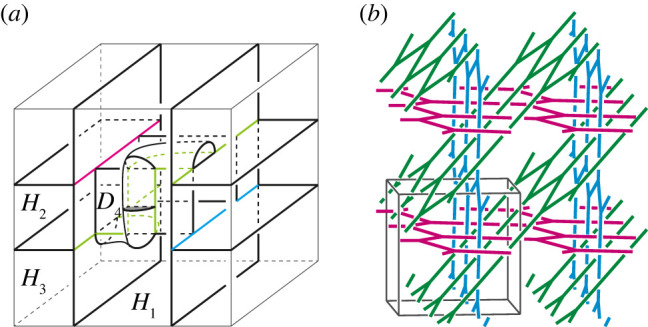
(*a*) A type-(2,2,2) handlebody decomposition of $T^{3}$. (*b*) A 3hcb net that is the preimage by the universal covering map of the core of the handlebodies $H_{1}$, $H_{2}$ and $H_{3}$.

First, for each i, we take three meridian discs $D_{i1}$, $D_{i2}$ and $D_{i3}$ of the handlebody $H_{i}$ as shown in figure 16*a*–*c*. Each disc intersects the singular graph of P transversely exactly two points. Furthermore, any two different discs are disjoint. Hence, we can perform type-1 destabilizations along them. By this operation, we obtain a type-(2,2,2) handlebody decomposition of $T^{3}$ (see figure 17*a*). For simplicity, we denote each handlebody and the partition of the destabilized handlebody decomposition by the same symbol $H_{1}$, $H_{2}$, $H_{3}$ and P, respectively. Note that the preimage of the union of spines of $H_{1}$, $H_{2}$ and $H_{3}$ is isotopic to a *3hcb* net as shown in figure 17*b*. See [31] for examples of materials with this chemical framework. See also [32]. The destabilized net-like pattern is also a simple coloured tricontinuous pattern.

**Figure 18.**
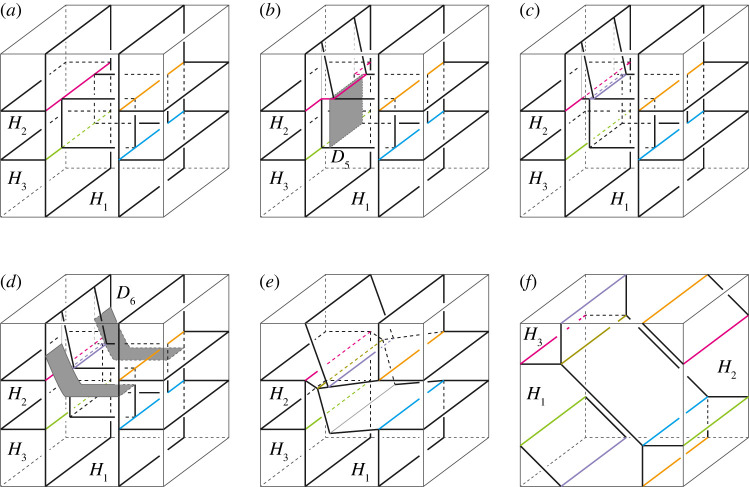
A sequence of type-1 destabilizations from the type-(2,2,1) handlebody decomposition to the type-(1,1,1) handlebody decomposition. (*a*) Type-(2, 2, 1), (*b*) a meridian disc *D*${\;}_{5}$ of *H*${\;}_{2}$, (*c*) type-(2, 1, 1), (*d*) a meridian disc *D*${\;}_{6}$ of *H*_1_, (*e*) type-(1, 1, 1), (*f*) the hexagonal honeycomb decomposition. (Online version in colour.)

For the type-(2,2,2) handlebody decomposition, we can perform a type-1 destabilization along a meridian disc $D_{4}$ of $H_{3}$ (figures 17*a* and 18*a*). The type of resulting decomposition is (2,2,1). Figure 18*a*–*e* illustrates a destabilization to the type-(2,2,1) handlebody decomposition, which produces a type-(1,1,1) handlebody decomposition. The type-(1,1,1) handlebody decomposition illustrated in figure 18*f* is the hexagonal honeycomb decomposition (figure 9*d*). ▪

## 7. Characterization of patterns

In this section, we will prove that bicontinuous patterns are unique. We will also show that simple, coloured, framed net-like patterns of type (1,1,1) are unique. On the other hand, we will provide two different simple coloured net-like patterns of type (1,1,1,1).

### (a) Bicontinuous patterns and Heegaard splittings of $T^{3}$

By definition, an n-continuous pattern consists of precisely n labyrinthine domains, and it is proper. Hence, by assigning a different colour to each domain, the pattern admits an n-colouring. In general, a frame of the pattern is not compatible with the colouring. However, by expanding the fundamental domain, we can obtain a frame compatible with the colouring. Then, by corollary 5.12, the pattern with the frame gives a proper type-$\left(g_{1},\ldots ,g_{n}\right)$ handlebody decomposition of $T^{3}$. Hence, the pattern is a framed net-like pattern of type $\left(g_{1},\ldots ,g_{n}\right)$. In particular, we note the following for each simple bicontinuous pattern and such a frame.

**Remark 7.1.**

Any simple bicontinuous pattern and its frame compatible with a colouring induce a Heegaard splitting of $T^{3}$.

By [33,34], Heegaard splittings of $T^{3}$ are determined by their Heegaard genera. Hence, we can prove the uniqueness of bicontinuous patterns.

**Theorem 7.2.**

*Any two simple bicontinuous patterns are equivalent.*

**Proof.**

Let $\left(\tilde{P},\pi \right)$ and $\left({\tilde{P}}^{\prime },\pi^{\prime }\right)$ be bicontinuous patterns of types (g,g) and $\left(g^{\prime },g^{\prime }\right)$, respectively. For the frame π, there exists a basis $\left\langle {\text{a}}_{1},{\text{a}}_{2},{\text{a}}_{3}\right\rangle$ of $R^{3}$ such that the translations $t_{i}$ defined by the vectors ${\text{a}}_{i}$ generate the covering transformation group. We denote by T a group generated by translations $t_{1}^{g^{\prime }-1}$, $t_{2}$ and $t_{3}$. Hence we have a covering map $\rho :R^{3}\to R^{3}/T≅T^{3}$. Since the Euler characteristic of $\tilde{P}/\rho$ is $\left(g^{\prime }-1\right)$-times that of $\tilde{P}/\pi$, the surface $\tilde{P}/\rho$ gives a Heegaard splitting of $T^{3}$ of genus $(g-1)(g^{\prime }-1)+1$. Similarly, we can take a covering map $\rho^{\prime }:R^{3}\to T^{3}$ so that ${\tilde{P}}^{\prime }/\rho^{\prime }$ also gives a Heegaard splitting of genus $(g-1)(g^{\prime }-1)+1$. Therefore, by using [34, Théorème] and proposition 5.16, the two simple bicontinuous patterns are equivalent. ▪

The Gyroid, the Schwartz D surface and the Schwartz P surface are famous triply periodic minimal surfaces that decompose $R^{3}$ into precisely two open components (see [35]), i.e. the surfaces are simple bicontinuous patterns. In [36, appendix], Squires *et al.* gave an isotopy from the Gyroid to the Schwartz D surface and the Schwartz D surface to the Schwartz P surface by an explicit formula. Note that theorem 7.2 is a generalization of the result but does not give a formula for transformation between patterns.

### (b) The uniqueness of framed patterns of type (1, 1, 1)

We consider the hexagonal honeycomb pattern introduced in example 5.7. Recall that its pattern admits a colouring and a frame compatible with it, as in figure 11*a*. The pattern induces the hexagonal honeycomb decomposition of $T^{3}$. Hence, the hexagonal honeycomb pattern with the frame is of type (1,1,1). By propositions 4.11 and 5.16, the hexagonal honeycomb pattern is a canonical model of simple coloured net-like patterns of type (1,1,1). Therefore, we have the following.

**Theorem 7.3.**

*Any simple, coloured, framed net-like pattern of type* (1,1,1) *is equivalent to the hexagonal honeycomb pattern*.

Note that a simple three-coloured net-like pattern whose labyrinthine nets consist of lines is not necessarily equivalent to the hexagonal honeycomb pattern in general (see example 7.4). Also, there are distinct simple coloured net-like patterns of type (1,1,1,1) (see example 7.5).

**Example 7.4.**

**Figure 19.**
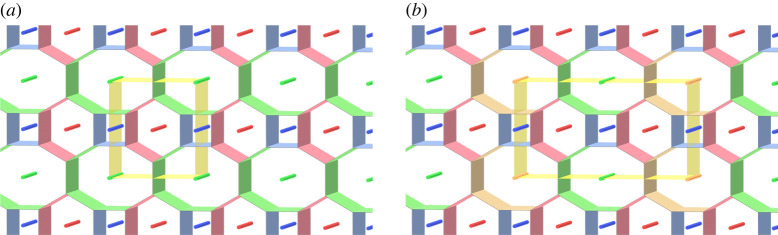
(*a*) A simple non-effectively coloured net-like pattern of type (1,1,1). (*b*) A simple coloured net-like pattern of type ([1,1],[1,1],1,1). The handlebody decomposition induced by the pattern contains two blue solid tori and two red solid tori. (Online version in colour.)

We consider a tessellation of the plane $R^{2}$ by three kinds of tiles: square, hexagon and eight-sided polygon. Figure 19 shows a net-like pattern induced by the tessellation. The left side (figure 19*a*) illustrates a framed net-like pattern of type (1,1,1) that is not coloured since eight-sided components are assigned to the same colour, and they are adjacent. On the other hand, the pattern admits a four-colouring (figure 19*b*). However, it is no longer type (1,1,1). This pattern is called [8,6,4;8,8,6] in [23, fig. 8(k)] and the colouring given there corresponds to a coloured net-like pattern of type ([1,1],[1,1],[1,1]).

**Example 7.5.**

**Figure 20.**
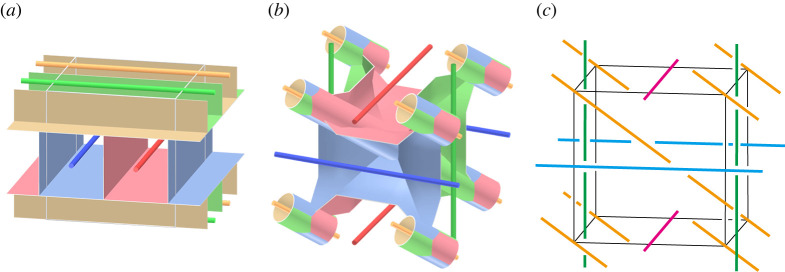
(*a*,*b*) Two framed simple coloured net-like patterns of type (1,1,1,1). (*c*) The labyrinthine nets of (*b*). (Online version in colour.)

Figure 20 illustrates two simple coloured net-like patterns, $\left({\tilde{P}}_{a},\rho_{a}\right)$ and $\left({\tilde{P}}_{b},\rho_{b}\right)$, of type (1,1,1,1) with a cubical fundamental domain, where $\rho_{a}$ and $\rho_{b}$ denote their frames compatible with the colourings, respectively. We can see the two patterns are not equivalent as follows. Let ${\tilde{X}}_{a}$ and ${\tilde{X}}_{b}$ be nets associated with ${\tilde{P}}_{a}$ and ${\tilde{P}}_{b}$. We consider the image ${\left(\iota_{a}\right)}_{*}(\pi_{1}(\rho_{a}({\tilde{X}}_{a})))$ and ${\left(\iota_{b}\right)}_{*}(\pi_{1}(\rho_{b}({\tilde{X}}_{b})))$, where $\iota_{a}$ and $\iota_{b}$ are the inclusion maps, respectively. By figure 20*a* ${\left(\iota_{a}\right)}_{*}(\pi_{1}(\rho_{a}({\tilde{X}}_{a})))$ is isomorphic to Z⊕Z. On the other hand, ${\left(\iota_{b}\right)}_{*}(\pi_{1}(\rho_{b}({\tilde{X}}_{b})))$ is isomorphic to Z⊕Z⊕Z by figure 20*b*. Hence, ${\tilde{P}}_{a}$ is not equivalent to ${\tilde{P}}_{b}$.

By theorem 7.3, any two simple coloured framed net-like patterns of type (1,1,1) are equivalent. However, simple coloured net-like patterns of type (1,1,1,1) are not unique.

The labyrinthine nets of these types of patterns are called cubic rod (cylinder) packings [37] or weavings [38]. Many of those structures do not correspond to simple coloured net-like patterns.

## Data Availability

This article has no additional data.
